# Development of the Basel Version of the Awareness of Social Inference Test – Theory of Mind (BASIT‐ToM) in healthy adults

**DOI:** 10.1111/jnp.12290

**Published:** 2022-09-21

**Authors:** Marianne Jarsch, Olivier Piguet, Manfred Berres, Constantin Sluka, Anna Semenkova, Reto W. Kressig, Andreas U. Monsch, Skye McDonald, Marc Sollberger

**Affiliations:** ^1^ Memory Clinic University Department of Geriatric Medicine Felix Platter Basel Switzerland; ^2^ Faculty of Psychology University of Basel Basel Switzerland; ^3^ School of Psychology and Brain and Mind Centre The University of Sydney Sydney New South Wales Australia; ^4^ Faculty of Mathematics and Technology University of Applied Sciences Koblenz Koblenz Germany; ^5^ Department of Clinical Research University of Basel and University Hospital Basel Basel Switzerland; ^6^ University of New South Wales Psychology Sydney New South Wales Australia; ^7^ Department of Neurology University Hospital Basel and University of Basel Basel Switzerland

**Keywords:** dynamic stimuli, healthy subjects, social cognition, test development, theory of mind

## Abstract

Impairments of Theory of Mind (ToM) abilities occur in a wide range of brain disorders. Therefore, reliable and ecologically valid examination of these abilities is a crucial part of any comprehensive neuropsychological assessment. An established and ecologically valid, English‐language test identifying deficits in ToM abilities is “The Awareness of Social Inference Test – Social Inference Minimal (TASIT‐SIM)”. However, no comparable German‐language ToM test currently exists. In this study, we aimed to develop the first German‐language adaption of TASIT‐SIM in healthy adults. We selected 13 scenes [four scenes per message type (i.e*.*, honesty, simple sarcasm, paradoxical sarcasm) and one practice scene] out of the 30 TASIT‐SIM scenes. In collaboration with a film institute, we filmed each scene at three different intensities. These intensity version scenes were then administered to 240 healthy adults, equally distributed in sex and age, ranging from 35 to 92 years. By applying Rasch analysis, we selected intensity versions that showed neither floor nor ceiling effects in the majority of ToM questions in participants whose ToM abilities were in the medium range. In conclusion, we have developed the first German‐language adaption of TASIT‐SIM, i.e*.*, the “Basel Version of the Awareness of Social Inference Test – Theory of Mind (BASIT‐ToM)”. The BASIT‐ToM incorporates the strengths of TASIT‐SIM, while overcoming its limitations such as inconsistencies in cinematic realization and ceiling effects in healthy participants. Next, the BASIT‐ToM needs to be validated in healthy people and clinical populations.

## BACKGROUND

Theory of Mind (ToM) – the adoption of cognitive (cognitive ToM) and affective (affective ToM) perspectives of others (Henry et al., 2016) – is a critical social cognitive skill that enables us to interact successfully with others (Kennedy & Adolphs, 2012). Adoption of another person's perspective requires both the ability in interpreting the other person's linguistic and paralinguistic characteristics (e.g., facial expressions, gestures and/or prosody) and distinguishing them from one's own (Henry et al., 2016). While cognitive ToM enables people to draw inferences about cognitive mental states such as thoughts and beliefs of another person, affective ToM enables people to infer affective mental states such as emotions from another person (Henry et al., 2016).

Apart from the distinction between cognitive and affective ToM, ToM can also be subdivided into so‐called orders. In general, first‐ and second‐order ToM tasks are assessed in clinical studies (Amodio & Frith, 2006; Canty et al., 2015; Castelli et al., 2011; Wang et al., 2018). First‐order ToM enables a person to draw social inferences about another person (e.g., first‐order cognitive ToM: Person A thinks that person B wants to quit his job; Meijering et al., 2011). Forming a second‐order ToM, however, is cognitively more complex as it requires higher‐level‐reasoning (Canty et al., 2017) and enables a person to mentalize social inferences that another person draws about another person (e.g., second‐order affective ToM: Person A thinks that person B feels sorry for person C as person B seems to perceive person C as sad; Meijering et al., 2011).

Lastly, apart from content and degree of cognitive complexity within the concept of ToM, ToM can be conceptualized as a continuum that ranges from no mentalization through to hypomentalization, adequate mentalization and finally hypermentalization (Abu‐Akel & Bailey, 2000). Whilst hypomentalizing can be seen as reduced mentalization, hypermentalization can be described as a kind of overattribution, in which additional mental hypotheses are generated, that cannot be directly deduced from the social situation (Abu‐Akel & Bailey, 2000; Frith, 2004). In other words, people with hypermentalization mentalize, but in an excessive and inadequate way. When examining ToM in patients with neuropsychological disorders, one needs to be aware of the continuum of ToM, as disturbances in ToM vary depending on the brain disorders. For example, patients with Asperger's syndrome, euthymic bipolar disorder, behavioural variant frontotemporal dementia or schizophrenia with negative symptoms tend to hypomentalize (Bora et al., 2015; Canty et al., 2017; Dziobek et al., 2006; Montag et al., 2010), whereas patients with borderline personality disorder or patients with schizophrenia with positive symptoms tend to hypermentalize (Canty et al., 2017; Sharp et al., 2011).

Deficits in ToM occur in a wide range of brain disorders, including developmental, neurological and psychiatric disorders (for review see Cotter et al., 2018). Given the high occurrence and clinical relevance of ToM deficits, the Diagnostic and Statistical Manual for Mental Disorders, Fifth Edition (DSM‐5; American Psychiatric Association, 2013) requires the assessment of ToM as a subdomain of social cognition for any comprehensive diagnosis of a neurocognitive disorder. To meet this goal, sensitive and reliable tests with high construct validity that assess different aspects of ToM within a practicable timeframe in clinical populations are needed. In addition, performance on tests of ToM should ideally predict the examined person's ability to adopt cognitive and affective perspectives of others in real life. In this regard, dynamic, realistic and socially relevant stimuli (e.g., film scenes) may meet this demand more than static stimuli do.

In terms of tests that contain dynamic, socially relevant stimuli validated for German‐speaking patients, only the Movie for the Assessment of Social Cognition (MASC; Dziobek et al., 2006) is available, to the best of our knowledge. In this test, a fifteen‐minute long film is shown about four adolescents who meet for a cooking and games evening. The film is interrupted 45 times. Each time, participants are asked questions about the interactions that have just been shown, which relate to emotions, thoughts and intentions of the protagonists. It has been shown that the MASC is a reliable, ecologically valid test which has proved sensitive to detect ToM deficits in different brain disorders such as Asperger's syndrome (Dziobek et al., 2006), personality disorder (Sharp et al., 2011) or schizophrenia (Martinez et al., 2017). However, given its quite challenging cognitive design (Dziobek et al., 2006; Pottgen et al., 2013), the test seems little suitable for patients with deficits in multiple cognitive domains such as patients with neurodegenerative diseases. In addition, the topic of the protagonists' interactions seem more of interest for young adults (for whom the test was primarily developed) than for other age groups, which limits its relevance in the general population. Lastly, test application takes a long time in subjects with cognitive disorders, e.g., 30–45 min in patients with mild to moderate traumatic brain injuries [J. Quinting, personal communication, October 29, 2021; (Quinting et al., 2020)], which precludes its regular use in clinical settings. Therefore, a German‐language test based on dynamic, multimodal stimuli in social interactions that examines ToM in a less cognitively demanding way for the general adult population in a timely fashion is needed.

We decided to develop a German‐language adaption of “The Awareness of Social Inference Test – Social Inference Minimal” (TASIT‐SIM), an established and ecologically valid, English‐language test identifying deficits in ToM abilities (McDonald et al., 2003). TASIT‐SIM consists of two test sets of 15 brief film scenes showing simple but realistic day‐to‐day social interactions. By use of four questions per scene, ToM abilities can be assessed based on the comprehension of intentions, beliefs, meanings and emotions in either sarcastic or honest conversational messages. TASIT‐SIM was designed as a criterion‐based test that can identify clear deficits in ToM abilities in adults (McDonald et al., 2003). Indeed, TASIT‐SIM can discriminate between healthy individuals and patients with different brain disorders including neurodegenerative diseases (e.g., behavioural variant frontotemporal dementia (Kumfor et al., 2017), semantic variant primary progressive aphasia (Rankin et al., 2009), progressive supranuclear palsy (Ghosh et al., 2012)), multiple sclerosis (Genova & McDonald, 2020), traumatic brain injuries (McDonald et al., 2017) and psychiatric disorders such as schizophrenia or bipolar disorder (Quidé et al., 2020). It has adequate reliability and there is evidence for construct and ecological validity (for review see McDonald, 2012; McDonald et al., 2004).

TASIT‐SIM, however, contains limitations that need to be addressed (see also Jarsch et al., 2022). First, because of its duration, the test does not lend itself to be easily used in clinical settings (Henry et al., 2014; Westerhof‐Evers et al., 2014). Second, the social interactions often appear unrealistic due to the high intensity of paralinguistic features and somewhat outdated appearances of actors, potentially biasing participants' capacity for ToM judgements. Third, the high intensity of the paralinguistic features result in near ceiling scores in the majority of healthy people (McDonald et al., 2006, 2015), preventing the detection of subtle ToM deficits. Finally, questions after each scene were not explicitly developed to evaluate different ToM concepts (i.e., first‐/second‐order ToM, cognitive/affective ToM, hypermentalization), but to evaluate overall ToM ability, which hampers the evaluation of these different aspects of ToM.

In this study, we created an adapted German‐language version of TASIT‐SIM with professional actors in collaboration with a film institute and administered it to 240 healthy participants to select the scenes that showed neither floor nor ceiling effects in the majority of ToM questions in participants whose ToM abilities were in the medium range. The adapted German‐language version was named the “Basel Version of the Awareness of Social Inference Test‐Theory of Mind (BASIT‐ToM)”.

## METHODS

### The Awareness of Social Inference Test (TASIT) – Social Inference Minimal (SIM) and Social Inference Enriched (SIE)

TASIT – Social Inference Minimal (SIM) and TASIT – Social Inference Enriched (SIE) are parts of TASIT (McDonald et al., 2003). TASIT comprises three parts, i.e., TASIT – Emotion Evaluation Test (TASIT‐EET), TASIT‐SIM and TASIT‐SIE. TASIT‐EET examines the ability to recognize basic emotions in social situations, whereas TASIT‐SIM and TASIT‐SIE examine the ability to make ToM judgements in social interactions (McDonald et al., 2003). The adapted German‐language version of TASIT‐EET, called the “Basel Version of the Awareness of Social Inference Test – Emotion Recognition” has been published previously (Jarsch et al., 2022).

TASIT‐SIM examines the ability to make ToM judgements based on linguistic and paralinguistic cues (e.g., gestures, facial expressions, prosody) in short, realistic, everyday dialogue interactions with either honest or sarcastic content and minimal contextual information (i.e., no information provided other than that presented in the short interaction). In honest exchanges, the paralinguistic features of the speaker are congruent with the literal message allowing the meaning to be inferred directly. In sarcastic statements, the paralinguistic features are incongruent with the spoken text. If this incongruency cannot be perceived, statements might be perceived as honest, meaningless, or bizarre (McDonald et al., 2003). In the SIM, sarcasm is subdivided into simple sarcasm (i.e., the literal message could be misinterpreted as honest) and paradoxical sarcasm (i.e., the literal message only appears meaningful if the sarcastic meaning is recognized).

TASIT‐SIE comprises a set of scenes showing dialogue interactions with either sarcastic messages or lies. As in the SIM, viewers have to perceive and interpret paralinguistic features of the speaker/s to identify the meaning of the literal message. In contrast to the SIM, scenes of the SIE provide additional information to the viewer about the true belief of the speaker or the true state of affairs by presenting visual or auditory/verbal cues before or after the interaction scene.

Two parallel test versions, i.e., form A and form B, exist for both SI‐forms. The SIM comprises 15 scenes [five scenes per three different message types (i.e., honesty, simple sarcasm, and paradoxical sarcasm)]. The SIE comprises 16 scenes [eight scenes per two different message types (i.e., sarcasm, lie)]. The scenes last 15–60 s and are administered in a quasi‐randomized order.

In both SI‐forms, ToM ability is assessed with four questions: one about the belief (thinking question), one about the intention (doing question), one about the message (saying question) and the last one about the emotion (feeling question) of the main actor who behaves either honestly or sarcastically, or who lies. Participants can answer each question with “yes”, “no”, or “do not know”.

The SIM and the SIE were developed to differentiate between cognitively healthy people “with average social skills” and individuals with clear ToM deficits (McDonald et al., 2003). Accordingly, actors were asked to act each scene in an exaggerated fashion such that the majority of healthy people should be able to answer the four questions correctly (McDonald et al., 2003).

### Development of the BASIT‐ToM scenes

We decided to choose only one of the SI‐forms as they differ only in their richness of contextual information, and both assess ToM similarly. Indeed, factor analysis conducted on data collected in patients with acquired brain injuries and healthy individuals demonstrated the presence of a single factor underlying both SI‐forms (Honan et al., 2016). As the BASIT‐ToM is aimed for use in patients with cognitive deficits, we favoured SIM over SIE, as it likely requires less cognitive capacities than SIE given its lower amount of contextual information. SIE comprises additional camera shots on the true state of affair by mean of prologues or epilogues, whereas SIM does not. Arguably, however, by leaving out SIE, which contains more contextual information than SIM, we may not assess fully the brain regions associated with contextual adjustment in ToM (Lavoie et al., 2016). Accordingly, one may wish to consider adapting the SIE scenes in a comparable way to the SIM scenes depending on the patient groups to be investigated and the research questions.

We named the German‐language adaption of TASIT‐SIM, the BASIT‐ToM. In the following, we present the development of the BASIT‐ToM for which we took a similar approach as for the BASIT‐ER (Jarsch et al., 2022).

#### Selection of the scenes from TASIT‐SIM

One test form was developed, rather than two, given negligible practice effects on TASIT‐SIM scores (McDonald et al., 2003). First, we selected 13 film scenes (4 × 3 message types for test scenes and one practice scene) from the 30 TASIT‐SIM scenes (15 form A scenes and 15 form B scenes; McDonald et al., 2003) based on 12 evaluation criteria that focus on different aspects of cinematographic quality, target group fit and cinematic feasibility (see Appendix A1). Three raters (MJ, MS, and a master's degree psychology student) rated each scene independently, and then agreed on a joint rating and selected the 13 scenes in consensus (see Appendix B: Tables B1.1–B1.3). The selected TASIT‐SIM scenes are depicted in the Appendix B: Table B2.

#### Conceptualization of the BASIT‐ToM scenes

We developed the conceptualization of the scenes and the cinematographic realization in collaboration with the film production company East End Film GmbH (Germany; https://www.eastendfilm.de) and adapted TASIT‐SIM as follows:

##### Screenplay

First, the transcription of TASIT‐SIM scenes (Westerhof‐Evers et al., 2014) was adapted to the German language without major changes of content. In three scenes (i.e., A9, A10, A13), we deleted text passages that either lengthened the scene without providing additional information or made it too difficult to understand. In two scenes (i.e., A11, B15), we supplemented the script with short sentences to achieve a more realistic conversation. Second, as in the BASIT‐ER (Jarsch et al., 2022), we omitted naming the actors to exclude any potential bias associated with naming. All scenes were cast with one woman and one man. BASIT‐ToM screenplays and description of the main changes in the text are presented in the Appendix C.

##### Cast and acting of the actors

We cast the scenes with four female and four male, middle‐aged, professional German‐speaking actors. Each actor portrayed a message type (i.e., honesty, simple sarcasm or paradoxical sarcasm) only once to avoid any actor‐specific associations to a message type. Each pair of actors played only one scene together to omit any potential biases due to varying role relationships between actors. Actors' sex were equally distributed across the different message types.

To make the scenes appear as realistic as possible, both actors in the scene acted in a realistic way. This type of acting differs from TASIT‐SIM in which the conversation partners act in a neutral way (i.e., the conversation partner does not seem to perceive the main actor's sarcasm). In real life, however, one would not expect another person to react in a neutral way to someone who is very sarcastic. Indeed, the conversation partner's neutral behaviour may confuse a viewer with preserved ToM capacity. The distribution of the actors to each of the 13 scenes is presented in the Appendix D.

##### Realism of scenes and camera work

We placed emphasis on a high degree of realism on the film scenes through realistic set designs either at office or at home backgrounds. All scenes were filmed as medium shots. Medium shots focus on the person in the scene while still showing some environment. Scenes were shot in 4K‐resolution.

##### Three intensity levels of the portrayed paralinguistic cues

*A*ctors were asked to portray the paralinguistic cues at three different levels of intensity (i.e., low, medium, and high). Text and set design remained identical at the three intensity versions. Background information was written for each scene and each intensity to help actors portraying the message types. After the shooting, MJ, MS and six master's degree psychology students selected the best scenes for each intensity by consensus. One exemplary scene (scene#4 of the message type paradoxical sarcasm) at low, medium and high levels of intensity, can be found at https://figshare.com/s/5ca4ff99790fcc5866db.

Background information for each BASIT‐ToM scene is described in the Appendix E.

#### Adaption of the questions of the BASIT‐ToM

In TASIT‐SIM, four kinds of questions were originally asked tapping thoughts, intentions, feelings and meanings of the speakers. While belief and intention questions, in particular, were originally designed to tap first‐order and second‐order ToM respectively, they varied in the extent to which this was successful. This was especially the case for the intention questions, which often asked for understanding of motives rather than beliefs. Other question types also tapped ToM to varying degrees (S. McDonald, personal communication, November 15, 2021; McDonald et al., 2003).

We decided to categorise existing questions as to whether they explicitly referred to (1) affective or (2) cognitive ToM and whether this was (3) first‐order or (4) second‐order. We also reworded several first‐order ToM questions and created additional second‐order ToM questions to get a more balanced representation of first‐ and second‐order questions. We were able to reword these questions because conversation partners act, in contrast to TASIT‐SIM, in a realistic way in the BASIT‐ToM scenes (see above “Cast and acting of the actors”). The generation of new second‐order ToM questions resulted in a more balanced proportion of first‐ and second‐order ToM questions [i.e., 65% first‐order ToM questions (first‐order cognitive ToM: *n =* 17, first‐order affective ToM: *n* = 17), 35% second‐order questions (second‐order cognitive ToM: *n =* 8, second‐order affective ToM: *n =* 10)]. First‐ and second‐order ToM and cognitive and affective ToM were partly balanced within and between the three message types, i.e., honesty: 19% first‐order cognitive ToM, 13% second‐order cognitive ToM, 44% first‐order affective ToM, 25% second‐order affective ToM; simple sarcasm: 38% first‐order cognitive ToM, 25% second‐order cognitive ToM, 25% first‐order affective ToM, 12% second‐order affective ToM; paradoxical sarcasm: 38% first‐order cognitive ToM, 6% second‐order cognitive ToM, 31% first‐order affective ToM, 25% second‐order affective ToM. Importantly, the distribution of ToM types may undergo further refinement, depending on the results of the planned validation study. We will also consider combining simple and paradoxical sarcasm scores that may result in a more balanced distribution of ToM types within and between message types. Taken together, we assume that separate analyses of ToM types will be feasible in the final BASIT‐ToM version. This is critical with regard to the future use of the BASIT‐ToM in clinical samples. Scores reflecting different ToM abilities, which partly reflect different neuroanatomic substrates (Corradi‐Dell'Acqua et al., 2020; Fortier et al., 2018; Poletti et al., 2012; Ryan et al., 2017), will likely better discriminate between brain diseases (Lancaster et al., 2019; Poletti et al., 2012; Rossetto et al., 2018) than an overall ToM score.

Unlike other ToM tests (e.g., MASC (Dziobek et al., 2006), Virtual Assessment of Mentalising Ability (VAMA; Canty et al., 2015)), the BASIT‐ToM contains multiple mentalizing questions per scene. Using normalization, we will be able to assess a participant's mentalization ability, ranging from no mentalization, to reduced mentalization (i.e., hypomentalization) to adequate mentalization. This approach, however, says nothing about hypermentalization (i.e., overattribution of another person's cognitive and/or affective mental states), which is why we created a hypermentalization question (hyperToM) for each scene. The hypermentalization question may be useful to discriminate between different clinical populations with behavioural disorders. For example, individuals diagnosed with schizophrenia who experience positive symptoms tend to hypermentalize (Canty et al., 2017), whereas patients with behavioural variant frontotemporal dementia, another syndrome associated with behavioural disorders, but due to neurodegeneration (Rascovsky et al., 2011), hypomentalize or are even unable to mentalize (Bora et al., 2015).

In TASIT‐SIM, participants could answer each question with “yes”, “no”, or “do not know” (McDonald et al., 2003). We reduced the three response alternatives to a forced‐choice paradigm, namely “yes” and “no”, as we considered the risk of people with cognitive disorders choosing the “do not know” option due to uncertainty or little motivation to be greater than the problem of guessing probability of 0.5 per question. Moreover, we reworded some questions to get a more balanced proportion of “yes” and “no” answers per scene to avoid any content‐independent tendency towards “yes” or “no” answers, respectively. For illustration, you find the adaption of a first‐order, cognitive TASIT‐SIM question into a second‐order, affective BASIT‐ToM question, as well as a hypermentalization question in Table 1.

**TABLE 1:** Example of an adaption of a first‐order, cognitive TASIT‐SIM question into a second‐order, affective BASIT‐ToM question as well as an example of a hypermentalization question

	Question	ToM type	ToM order	Correct answer
TASIT‐SIM	Does Michael think she took it easy on the weekend?	cogn	1st	no
BASIT‐ToM	Does he think it was okay for her working all weekend?	aff	2nd	yes
	Is he asking her about the report to provoke her?	hyperToM		no

Abbreviations: aff, affective ToM; BASIT‐ToM, Basel Version of the Awareness of Social Inference Test‐Theory of Mind; cogn, cognitive ToM; hyperToM, hypermentalization; TASIT‐SIM, The Awareness of Social Inference Test‐Social Inference Minimal; ToM, Theory of Mind.

All questions of each BASIT‐ToM scene and the respective TASIT‐SIM scene with representation of ToM types, ToM orders and correct answers, as well as the hypermentalization questions are found in the Appendix F. The main differences between BASIT‐ToM and TASIT‐SIM scenes are described in the Appendix G.

### Selection of the BASIT‐ToM intensity version scenes for use in clinical populations

#### Participants

Next, we administered the BASIT‐ToM intensity version scenes to 240 cognitively and mentally healthy Central European subjects (50% women) with mother language (Swiss)‐German to select scenes neither showing floor nor ceiling effects. We opted for a large age range to make the results applicable to the general adult population. We defined five age groups (35–44, 45–54, 55–64, 65–74, >75) to achieve an even distribution of sex by age. Each group consisted of 48 participants (50% women; at maximum three participants were at the same age). Participants were included if they met the following inclusion criteria: 35 years of age or older, total education of seven years or greater, German and/or Swiss German as first language, and self‐report of good health. Exclusion criteria were conditions with potential negative influence on the test results, including signs of depressive mood (i.e., scoring ≥10 points on the Beck Depression Inventory (Beck et al., 1961) for individuals below 65 years, or ≥5 on the Geriatric Depression Scale (Yesavage & Sheikh, 1986) for individuals aged ≥65 years), cognitive deficits [i.e., Montreal Cognitive Assessment (Nasreddine et al., 2005) score below the demographically‐adjusted, fifth percentile for cognitively healthy individuals (Thomann et al., 2018)], systemic or brain diseases, psychiatric disorders according to the ICD‐10 criteria, traumatic brain injury, chronic pain, history or current regular intake of any psychoactive drugs (except benzodiazepines for sleep), general anaesthesia within the last three months and severe sensory and/or motor deficits. The study was approved by the local ethics committee and all participants provided written informed consent.

#### Application of the BASIT‐ToM scenes

BASIT‐ToM scenes and the test paradigm were displayed on a monitor with a diagonal size of 24 inches, a 16:10 aspect ratio and a 1920:1200 display resolution using Python 2.7 (Peirce, 2007) and PsychoPy 1.84.2 package (Peirce, 2009). Details of the programming and data storage are described in the Appendix G.

Before the test started, participants were provided with standardized instructions by the examiner. Then, they read the test instructions on the computer monitor. Next, one practice scene was shown to familiarize the participant with the procedure and to clarify any potential questions. The 12 test scenes were then administered in a pseudo‐randomized order (not the same message type twice in a row). Each message type [i.e., honesty (H), simple sarcasm (sS), paradoxical sarcasm (pS)] was shown in four scenes (3 × 4). Of the four scenes of each message type, participants watched one scene at low intensity, another scene at medium intensity, another scene at high intensity, and another scene at either low, medium, or high intensity. For example, participant#1 watched H#1 at low intensity, H#2 at medium intensity, H#3 at high intensity, and H#4 at low intensity, whereas participant#2 watched H#1 at medium intensity, H#2 at high intensity, H#3 at low intensity, and H#4 at medium intensity. As there were three intensity levels per scene, each intensity version scene was watched by 80 participants. Each participant watched 13 intensity version scenes, i.e., one practice scene at a given intensity +12 test scenes at given intensities [3(message types) × 4(intensity versions)].

By taking the approach that each participant watches only one intensity per scene, we avoided potential biases on responses arising from watching a scene that you have seen and rated previously at a different intensity.

Following each scene, participants were required to answer the four ToM questions and the hyperToM question with “yes” or “no.” There was no time limit for answering the questions.

If participants were uncertain about a scene's content, they could rewatch the respective scene. They could rewatch a scene as many times as they wanted before answering. We noted the number of times a scene was watched. By noting it, we learned, in addition to the participants' ToM responses, about potential difficulties in the understanding of the respective scene content. We considered this information critical given the fact that we had developed new scenes, albeit based on TASIT‐SIM templates.

Similarly, participants could change their answers within a question block. An exemplary representation of the BASIT‐ToM computer‐based application process is depicted in Appendix H.

### Data analysis

The aim of the data analysis of the 39 intensity version scenes [3 (message types) × 4 (scenes) × 3 (intensities) = 36 intensity versions + 3 practice scene intensity versions] was to select one intensity version per scene that resulted in neither floor nor ceiling performance in participants whose ToM abilities were in the medium range for as many of the respective four ToM questions as possible. We prioritized the four ToM questions over the hyperToM question in our analysis approach as we aimed primarily for scenes that are adequate to detect decreased ToM ability. To achieve this type of scene selection, we took the following four‐steps approach:

#### Step 1

We conducted Rasch analysis (Rasch, 1960) by applying the R ltm package (Rizopoulos, 2006) with RStudio (R Studio Team, 2016) in R version 3.5.1 (R Core Team, 2020) to analyze the relations between the estimated participants' ToM ability parameters and the probabilities of correct answering the four ToM questions of the respective intensity version.

In each of the three message type analyses, we included the respective twelve intensity versions (4 scenes per message type × 3 intensities). In the practice scene analysis, we included the respective three intensity versions. Separate analyses were carried out for each message type intensity version scene and each practice scene intensity version. In order to check the model fit of the data, we conducted the ltm package parametric Bootstrap goodness‐of‐fit test using Pearson's χ^2^, based on 201 data sets (the original data set plus 200 simulated datasets). The scenes that showed model fit were selected for further analysis.

#### Step 2

Based on the graphical assessment of the item characteristic curve (ICC) plots of the intensity versions showing model fit, we aimed to choose one intensity per message type scene and one intensity for the practice scene that predicted a correct answering probability between 0.5 and 0.8 for the majority of the respective four ToM questions in participants with a medium ToM ability around 0 [−0.5, 0.5].

#### Step 3

Next, we examined the 95% confidence intervals (CIs) of the estimated difficulties of the selected intensity versions in relation to the required difficulty interval. CIs were calculated as estimate ±1.96 × standard error of estimate. The required difficulty was calculated according to the scene selection criteria, mentioned above at step 2 (i.e., probability between 0.5 and 0.8 in participants with a medium ToM ability around 0). This step allowed us to check whether the CI of the estimated difficulties of the selected scenes covered the required difficulty interval. In addition, we evaluated whether systematic differences in difficulty were present between first‐ and second‐order ToM questions and between cognitive and affective ToM questions, respectively. For this, we checked whether the estimated difficulty intervals of these questions overlapped (i.e., equal difficulties of the questions) or not (i.e., different difficulties of the questions).

#### Step 4

After completing scene selection, we ran separate Rasch analyses with each of the selected intensity versions by additionally including the respective hyperToM question to evaluate the probability of correct answering the hyperToM questions.

### Distribution of demographic variables between the selected intensity version scenes

As participants were randomly assigned to each intensity version scene and given the potential influence of demographic variables on ToM performance, we compared the mean scores and standard deviations of age and years of education as well as the sex ratio across the selected intensity version scenes.

#### Transparency and openness

In accordance with the Transparency and Openness Promotion (TOP; Nosek et al., 2015) and the Journal Article Reporting Standards‐Quantitative for non‐experimental designs (JARS‐Quant; Appelbaum et al., 2018), we have reported in detail how we developed the BASIT‐ToM stimulus material. The data that support the findings of this study are openly available in “figshare” at https://figshare.com/s/d7194f6f2f18082e623c.

## RESULTS

### Selection of the scenes

#### Step 1: Selection of intensity versions with an acceptable model fit

The parametric Bootstrap goodness‐of‐fit test showed an acceptable fit of the Rasch model (*p* > .05) for the analyses of the four ToM questions for 22 of the 39 intensity versions. ICC of the 22 intensity versions with an acceptable model fit are shown in Appendix J.

#### Step 2: Selection of intensity versions based on response probabilities

**FIGURE 1:**
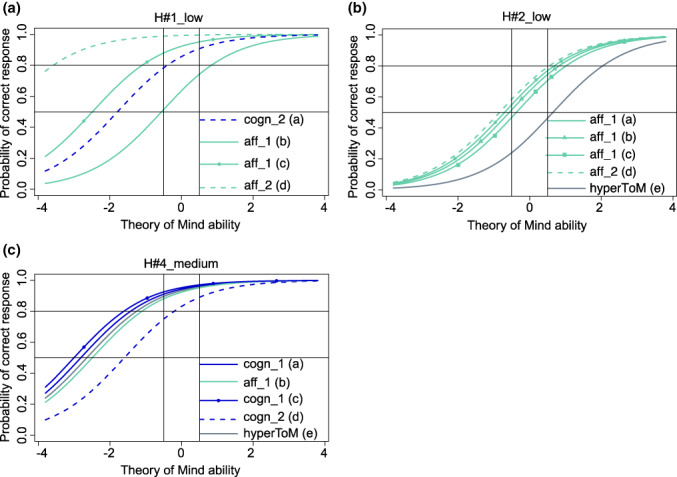
Item characteristic curves (ICC) of the theory of mind (ToM) and hypermentalization questions (hyperToM) of the three selected BASIT‐ToM scenes for message type honesty (i.e., a: Scene H#1_low, b: Scene H#2_low, c: Scene H#4_medium). ICC of the hyperToM question is only depicted in presence of a model fit. Scene code consists of a scene number (scene), a letter (message type, H = honesty), and the portrayed intensity (i.e., low or medium). Question code consists of abbreviations of type (i.e., cogn = cognitive ToM, aff = affective ToM, hyperToM = hypermentalization), order (i.e., 1 = first‐order ToM, 2 = second‐order ToM) of ToM, and question's identification letter (a‐d = ToM questions, e = hypermentalization question). Line with circles = ToM question of same type and order as another ToM question in the same panel; line with squares = ToM question of same type and order as two other questions in the same panel. BASIT‐ToM, Basel Version of the Awareness of Social Inference Test – Theory of Mind

**FIGURE 2:**
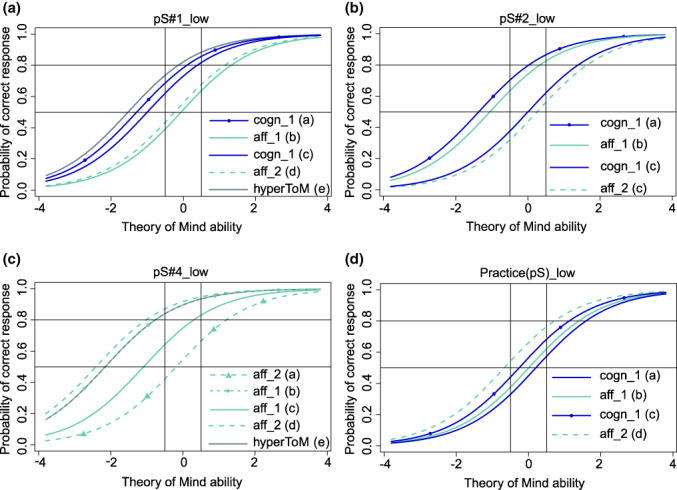
Item characteristic curves (ICC) of the theory of mind (ToM) and hypermentalization questions (hyperToM) of the four selected BASIT‐ToM scenes for message type paradoxical sarcasm [i.e., a: Scene pS#1_low, b: Scene pS#2_low, c: Scene pS#4_low, d: Practice (pS)_low]. ICC of the hyperToM question is only depicted in presence of a model fit. Scene code consists of a scene number (scene), a letter (message type, pS = paradoxical sarcasm), and the portrayed intensity (i.e., low). Question code consists of abbreviations of type (i.e., cogn = cognitive ToM, aff = affective ToM, hyperToM = hypermentalization) and order (i.e., 1 = first‐order ToM, 2 = second‐order ToM) of ToM, and question's identification letter (a–d = ToM questions, e = hypermentalization question). Line with circles or line with triangles = ToM question of same type and order as another ToM question in the same panel. Practice(pS_low) will be used as a test scene for paradoxical sarcasm in BASIT‐ToM, whereas PS#4_low will be used as practice scene. BASIT‐ToM, Basel Version of the Awareness of Social Inference Test – Theory of Mind

**FIGURE 3:**
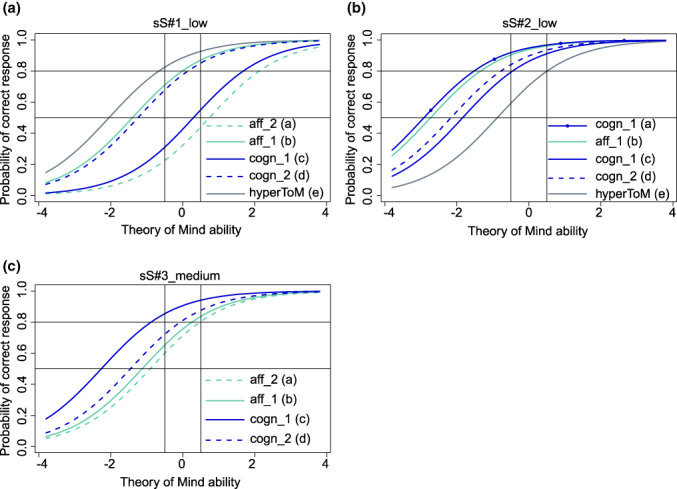
Item characteristic curves (ICC) of the theory of mind (ToM) and hypermentalization questions (hyperToM) of the three selected BASIT‐ToM scenes for message type simple sarcasm (i.e., a: Scene sS#1_low, b: Scene sS#2_low, c: Scene sS#3_medium). ICC of the hyperToM question is only depicted in presence of a model fit. Scene code consists of a scene number (scene), a letter (message type, sS = simple sarcasm) and the portrayed intensity (i.e., low or medium). Question code consists of abbreviations of type (i.e., cogn = cognitive ToM, aff = affective ToM, hyperToM = hypermentalization) and order (i.e., 1 = first‐order ToM, 2 = second‐order ToM) of ToM, and question's identification letter (a–d = ToM questions, e = hypermentalization question). Line with circles = ToM question of same type and order as another ToM question in the same panel. BASIT‐ToM, Basel Version of the Awareness of Social Inference Test – Theory of Mind

We selected 10 intensity versions [i.e., 3 intensity versions per message type (i.e., 3 × 3) and 1 intensity version for the future practice scene] from the 22 intensity versions that showed model fit of the Rasch model and predicted probabilities of a correct response between 0.5 and 0.8 for as many of the respective four ToM questions as possible in participants with a medium ToM ability around 0 [−0.5, 0.5] (see the H scenes in Figure 1a–c, the pS scenes in Figure 2a–d and the sS scenes in Figure 3a–c). All selected intensity versions are shown in Table 2, and the ToM types and orders of the questions associated with these intensity versions are shown in Table 3.

**TABLE 2:** Selected intensity version scenes for the BASIT‐ToM for further use in clinical populations

	Intensity version				
Message type	Scene 1	Scene 2	Scene 3	Scene 4	Scene 5
Honesty	Low	Low	–	Medium	n/a
Simple sarcasm	Low	Low	Medium	–	n/a
Paradoxical sarcasm	Low	Low	–	Low^a^	Practice (pS)_low^b^

Abbreviations: BASIT‐ToM, Basel Version of the Awareness of Social Inference Test‐Theory of Mind; n/a, not available; pS, paradoxical sarcasm.

^a^ pS#4_low (scene #4 containing paradoxical sarcasm at low intensity) will be used as practice scene in BASIT‐ToM.

^b^ Practice(pS)_low will be used as a test scene for paradoxical sarcasm in BASIT‐ToM.

**TABLE 3:** Types and orders of theory of mind of each question of the ten selected scenes

	ToM type and order				
Scene	a	b	c	d	e
H#1	cogn_2^nd^	aff_1^st^	aff_1^st^	aff_2^nd^	Hyper
H#2	aff_1^st^	aff_1^st^	aff_1^st^	aff_2^nd^	Hyper
H#4	cogn_1^st^	aff_1^st^	cogn_1^st^	cogn_2^nd^	Hyper
sS#1	aff_2^nd^	aff_1^st^	cogn_1^st^	cogn_2^nd^	Hyper
sS#2	cogn_1^st^	aff_1^st^	cogn_1^st^	cogn_2^nd^	Hyper
sS#3	aff_2^nd^	aff_1^st^	cogn_1^st^	cogn_2^nd^	Hyper
pS#1	cogn_1^st^	aff_1^st^	cogn_1^st^	aff_2^nd^	Hyper
pS#2	cogn_1^st^	aff_1^st^	cogn_1^st^	aff_2^nd^	Hyper
pS#4	aff_2^nd^	aff_1^st^	aff_1^st^	aff_2^nd^	Hyper
Practice (pS)	cogn_1^st^	aff_1^st^	cogn_1^st^	aff_2^nd^	Hyper

*Note*: ToM, Theory of Mind. Scene code consists of letters (i.e., H, honesty, pS, paradoxical sarcasm, sS, simple sarcasm) and the respective scene numbers. Question's identification letter = a–e (a–d = ToM questions, e = hypermentalization question); aff_1^st^, affective ToM first‐order; aff_2^nd^, affective ToM second‐order; cogn_1^st^, cognitive ToM first‐order; cogn_2^nd^, cognitive ToM second‐order; hyper, hypermentalization question.

The number of ToM questions that met the required response probabilities varied between the selected intensity versions. In 8 (80%) of the 10 selected intensity version scenes at least half of the questions met the required response probabilities, i.e., in 4 scenes (i.e., H#2_low, pS#1_low, pS#2_low, practice(pS)_low), the response probabilities were met by all four ToM questions; in 2 scenes (i.e., sS#1_low, sS#3_medium), they were met by three questions and in another 2 scenes (i.e., H#1_low, pS#4_low), they were met by two questions. In the H#4_medium and sS#2_low scenes, only one question met the required response probability. The response probabilities of the questions that were outside the required range were predicted higher (>0.8) apart from the affective second‐order question in sS#1_low, which was predicted lower (<0.5).

#### Step 3: Evaluation of the estimated difficulties of the ten selected intensity versions

**FIGURE 4:**
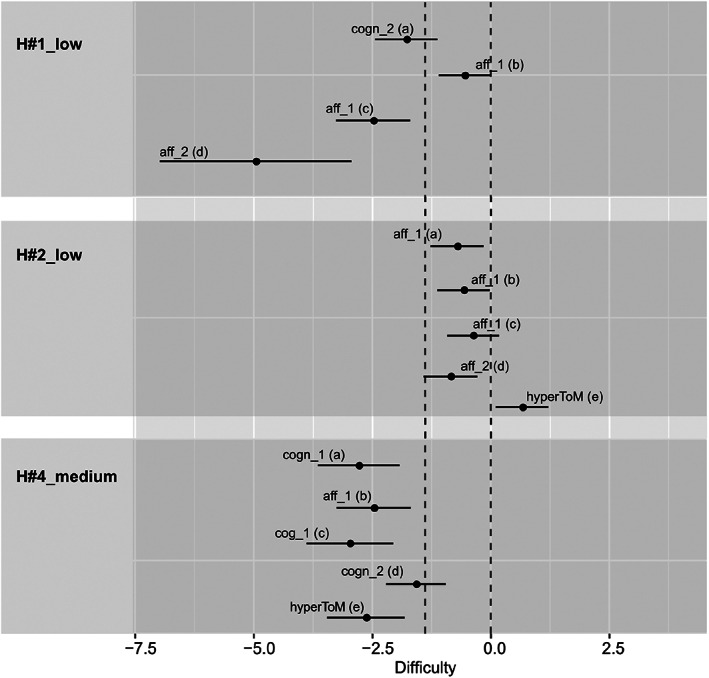
95% confidence intervals (CI) of the estimated difficulties of the theory of mind (ToM) and hypermentalization questions (hyperToM) of the three selected BASIT‐ToM scenes for message type honesty (i.e., a: Scene H#1_low, b: Scene H#2_low, c: Scene H#4_medium). CI of the hyperToM question is only depicted in presence of a model fit. Scene code consists of a scene number (scene), a letter (message type, H = honesty) and the portrayed intensity (i.e., low or medium). Question code consists of abbreviations of the type (i.e., cogn = cognitive ToM, aff = affective ToM, hyperToM = hypermentalization) and order (i.e., 1 = first‐order ToM, 2 = second‐order ToM) of ToM, and question's identification letter (a‐d = ToM questions, e = hypermentalization question). The dashed lines illustrate the required range of difficulty [−1.386, 0]. BASIT‐ToM, Basel Version of the Awareness of Social Inference Test – Theory of Mind

**FIGURE 5:**
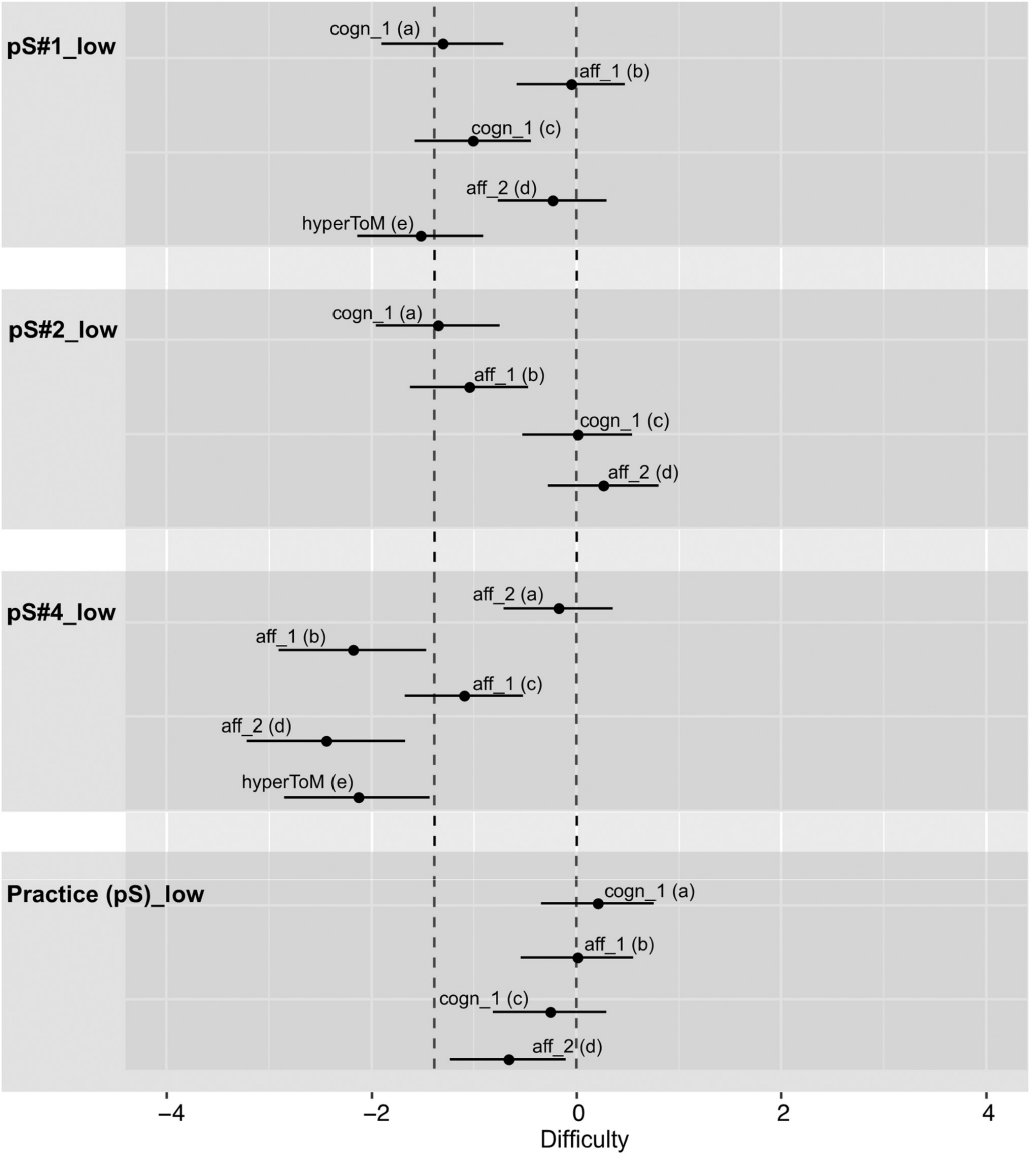
95% confidence intervals (CI) of the estimated difficulties of the theory of mind (ToM) and hypermentalization questions (hyperToM) of the four selected BASIT‐ToM scenes for message type paradoxical sarcasm [i.e., a: Scene pS#1_low, b: Scene pS#2_low, c: Scene pS#4_low, d: Practice (pS)_low]. CI of the hyperToM question is only depicted in presence of a model fit. Scene code consists of a scene number (scene), a letter (message type, pS = paradoxical sarcasm) and the portrayed intensity (i.e., low). Question code consists of abbreviations of the type (i.e., cogn = cognitive ToM, aff = affective ToM, hyperToM = hypermentalization) and order (i.e., 1 = first‐order ToM, 2 = second‐order ToM) of ToM, and question's identification letter (a–d = ToM questions, e = hypermentalization question). Practice(pS_low) will be used as a test scene for paradoxical sarcasm in BASIT‐ToM, whereas PS#4_low will be used as practice scene. The dashed lines illustrate the required range of difficulty [−1.386, 0]. BASIT‐ToM, Basel Version of the Awareness of Social Inference Test – Theory of Mind

**FIGURE 6:**
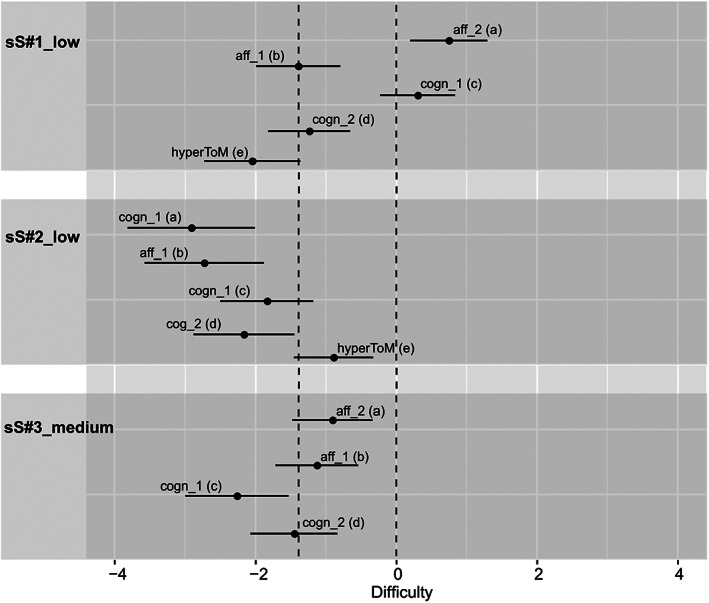
95% confidence intervals (CI) of the estimated difficulties of the theory of mind (ToM) and hypermentalization questions (hyperToM) of the three selected BASIT‐ToM scenes for message type simple sarcasm (i.e., a: Scene sS#1_low, b: Scene sS#2_low, c: Scene sS#3_medium). CI of the hyperToM question is only depicted in presence of a model fit. Scene code consists of a scene number (scene), a letter (message type, sS = simple sarcasm), and the portrayed intensity (i.e., low or medium). Question code consists of abbreviations of the type (i.e., cogn = cognitive ToM, aff = affective ToM, hyperToM = hypermentalization) and order (i.e., 1 = first‐order ToM, 2 = second‐order ToM) of ToM, and question's identification letter (a‐d = ToM questions, e = hypermentalization question). The dashed lines illustrate the required range of difficulty [−1.386, 0]. BASIT‐ToM, Basel Version of the Awareness of Social Inference Test – Theory of Mind

Next, we examined how well the 95% CI of the estimated difficulties of the four ToM questions of the selected intensity versions covered the required range of difficulty [i.e., −1.386,0 (minus the logits of 0.8 = −1.386, minus the logits of 0.5 = 0); see the H scenes in Figure 4, the pS scenes in Figure 5 and the sS scenes in Figure 6]. The estimated difficulties were considered within the required range of difficulty when at least half of the CI covered the required range. In two of the 10 selected scenes (20%; i.e., H#2_low, pS#1_low), all four questions were within the required range. In another two scenes (i.e., pS#2_low, practice(pS)_low), three of the four questions were within the required range, whereas one question was slightly above the required difficulty (too difficult). In three scenes (i.e., pS#4_low, sS#1_low, sS#3_low), two questions were within the required range. In pS#4_low, the other two questions were below the required difficulty (too easy), whereas in sS#1_low and sS#3_low the other two questions were above the required difficulty (too difficult). In one scene (i.e., H#1_low), one question was within the required range, whereas the other three questions were below the required difficulty (too easy). In two scenes (i.e., H#4_medium, sS#2_low), all questions were below the required difficulty (too easy).

Notably, we found no systematic differences in terms of difficulty between first‐ and second‐order ToM questions and between cognitive and affective ToM questions, respectively (Figures 4, 5, 6).

#### Step 4: Evaluation of the HyperToM questions in terms of model fit and response probabilities

Finally, we checked whether the model fit of each of the 10 selected intensity versions persisted after inclusion of the hyperToM question in the model and if yes, how well the 95% CI of the estimated difficulties of the hyperToM questions covered the required range of difficulty (i.e., −1.386,0). In six scenes (60%; i.e., H#2_low, H#4_medium, pS#1_low, pS#4_low, sS#1_low, sS#2_low), there was still a model fit. In two [i.e., pS#1_low (Figure 2a), sS#2_low (Figure 3b)] of these scenes, the response probability of the hyperToM question was within the required range of 0.5–0.8 (Figures 5 and 6); in three scenes [i.e., H#4_medium (Figure 1c), pS#4_low (Figure 2c), sS#1_low (Figure 3a)], it was above 0.8 [too easy, Figures 4, 5, 6] and in one scene [i.e., H#2_low (Figure 1b)], it was below 0.5 [too difficult, Figure 4]. A model fit was no longer present in four scenes (i.e., H#1_low, pS#2_low, practice(pS)_low, sS#3_medium).

### Number of times the selected intensity version scenes were watched

The majority of participants (83%) watched the selected intensity version scenes once only. Selected intensity version scenes were watched more than once as follows: twice: 16%, three times: 1%, and four times: <1%. None of the selected intensity version scenes was watched only once by all 80 participants. Percentages of scenes watched once only ranged from 58% (pS#2_low) to 91% (H#1_low, sS#1_low, sS#3_medium). For more details, please see Table K.1 in the Appendix K.

### Distribution of demographic variables between the selected intensity version scenes

We found similar distributions of the demographic variables across the selected intensity version scenes: For years of age, means ranged from 56.58 to 62.59 with standard deviations ranging from 13.34 to 16.34; for years of education, means ranged from 15.09 to 16.06 with standard deviations ranging from 2.52 to 3.15 and for sex, percentage of females ranged from 41% to 59%.

## DISCUSSION

In this study, we developed the BASIT‐ToM, the first German‐language TASIT‐SIM adaption and administered it to a sample of 240 healthy adults with a large age range. We adapted TASIT‐SIM to address some methodological and practical limitations of the original test, such as inconsistencies in cinematic realization, ceiling effects in healthy participants, and the long‐time of administration. In addition, we created a hypermentalization question for each of the BASIT‐ToM scenes.

We identified 10 scenes [1 practice item and 3 scenes apiece for the three message types (i.e., honesty, simple sarcasm and paradoxical sarcasm) that will comprise the BASIT‐ToM]. These scenes showed in general neither floor nor ceiling effects in the majority of ToM questions. As evidence of validity, we found that gradual changes in expressed intensities of either honest or sarcastic message types related to difficulties in ToM as measured by participants' correct answering probabilities.

Regarding the hypermentalization question, we were able to reliably analyse its response probabilities in six of the 10 scenes. These questions were in general easy to answer by the healthy participants, which is unsurprising given the fact that these participants are cognitively and mentally healthy. Future studies will show whether patients who hypermentalize such as schizophrenic patients with positive symptoms (Canty et al., 2017) or patients with borderline personality disorder (Sharp et al., 2011) will fail on the hypermentalization questions. In this regard, it is worth mentioning that BASIT‐ToM contains less contextual information than those tests [i.e., MASC (Dziobek et al., 2006), VAMA (Canty et al., 2015)] that were able to detect hypermentalization in these two patient groups. It will be interesting to investigate whether these patients do also hypermentalize when having watched BASIT‐ToM scenes, which contain little contextual information. If so, then the BASIT‐ToM would allow assessing not only decrease (hypomentalization) or loss (no mentalization) of ToM but also excessive ToM (hypermentalization). This is of high clinical value in the assessment of patients with neuropsychological disorders, because as mentioned above, some patient groups hypermentalize (Canty et al., 2017; Sharp et al., 2011), whereas for example patients with behavioural variant frontotemporal dementia, Asperger's syndrome, euthymic bipolar disorder, or schizophrenia with negative symptoms hypomentalize or do not mentalize at all (Bora et al., 2015; Canty et al., 2017; Dziobek et al., 2006; Montag et al., 2010).

Notably, we found no systematic differences in difficulty either between cognitive and affective questions or between first‐ and second‐order ToM questions. The absence of a difference in difficulty between first‐ and second‐order ToM questions may seem surprising as higher order ToM questions tend to be cognitively more demanding as shown among others by recruitment of more ToM‐associated brain regions in high‐order than low‐order ToM questions in cognitively healthy subjects (Lewis et al., 2017). Clinically, this seems to show up primarily in the healthy subjects' reaction times, but not in response accuracy (Lewis et al., 2017). In our paradigm, however, we measured the accuracy of responses rather than reaction times. Nevertheless, for individuals with cognitive deficits, for whom the test is designed for, it can be assumed that the higher cognitive load of the second‐order ToM questions will be reflected in the test scores.

Participants had the option to rewatch the scenes in case they had not understood the content of the scene. We set this option to get an idea about participants' understanding of the scene play. Notably, most participants did not use this option, which demonstrates the quality of the scene play and contents. Arguably, one of the selected intensity versions was watched at least twice by 42% of the participants. The majority of this scene's ToM questions, however, were within the required range of difficulty, indicating adequate understanding of the scene's content by the participants. We will reanalyse the scene's capacity in measuring ToM in the subsequent validation study with patients with neuropsychological disorders. As we speak of patients with neuropsychological disorders, who make up the target population for whom we designed the BASIT‐ToM, the opportunity to rewatch the scene before answering the questions to be sure about the scene content is critical. This way, we minimize the risk that low ToM test scores are due to cognitive dysfunction other than dysfunction in mentalizing. We agree that no “rewatch button” exists in real life. However, in real life, you are experiencing the unfolding of the scene, which likely facilities ToM ability.

Our study contains some limitations. First, in six of the selected scenes (i.e., H#1_low, H#4_medium, pS#4_low, sS#1_low, sS#2_low, sS#3_medium), some ToM questions were too easy to answer for participants with medium ToM abilities. Thus, these questions may not capture subtle deficits in ToM abilities. We will examine this in the subsequent validation study in a clinical sample and, if needed, remove scenes with too little discriminative power between patients with known ToM deficits such as patients with behavioural variant frontotemporal dementia (Bora et al., 2015; Henry et al., 2014) and healthy individuals.

Second, in four scenes (i.e., H#1_low, pS#2_low, practice(pS)_low, sS#3_medium), the inclusion of the hyperToM question in the data analysis resulted in a loss of model fit of the Rasch model. Likewise, these four questions seemed to be unclear to the participants, showing percentages of correct answers between 11% and 50%. Interestingly, all four questions referred to protagonists' affects (“Is he in love with her?,” “Can they not stand Simone?”), whereas the hyperToM questions with an adequate model fit referred to protagonists' intentions. The scenes' paralinguistic features and contextual information were probably insufficient to generate meaningful affective hyperToM questions. Based on these findings, we decided to limit ourselves to cognitive hyperToM questions in the BASIT‐ToM and, accordingly, will replace these four affective hyperToM questions by cognitive hyperToM questions.

Third, first‐, and second‐order ToM and cognitive and affective ToM were only partly balanced within and between the three message types. Importantly, however, the distribution of ToM types will undergo further refinement if needed, depending on the results of the planned validation study. We will also consider combining the simple and paradoxical sarcasm scores that may result in a more balanced distribution of ToM types within and between message types.

Finally, the large age range of our sample may be considered a limitation, given the evidence of an association between age and ToM (Henry et al., 2016). Similarly, sex and years of education may also influence performance. We therefore compared the distribution of the demographic variables between the selected intensity version scenes and found similar distributions. Accordingly, the influence of demographic variables on test performance appears unlikely. A related point, however, is that given the relatively high educational level of the participants, generalization of our findings to the general population needs to be done with caution.

Similar to the procedure used with the already published BASIT‐ER (Jarsch et al., 2022), we plan to validate the BASIT‐ToM both in a healthy population and in a clinical sample to examine its reliability, construct and ecological validity, sensitivity in detecting ToM deficits, and the potential influence of cognitive deficits on test performance. Thereby, we will score both, honest and sarcastic exchanges, and the different types and orders of ToM separately to evaluate whether this scoring approach improves discrimination between patients with different brain disorders compared to an overall ToM score. Likewise, we will evaluate the utility of hypermentalization questions to discriminate between patient groups. Based on the results of the validation study, we will set the final version of the BASIT‐ToM for later use in clinical settings.

## AUTHOR CONTRIBUTIONS

**Marianne Jarsch:** Conceptualization; data curation; formal analysis; methodology; visualization; writing – original draft; writing – review and editing. **Olivier Piguet:** Writing – review and editing. **Manfred Berres:** Formal analysis; methodology; writing – review and editing. **Constantin Sluka:** Data curation; resources; software; writing – review and editing. **Anna Semenkova:** Formal analysis; writing – review and editing. **Reto W. Kressig:** Writing – review and editing. **Andreas U. Monsch:** Writing – review and editing. **Skye McDonald:** Writing – review and editing. **Marc Sollberger:** Conceptualization; formal analysis; funding acquisition; investigation; methodology; project administration; resources; supervision; validation; writing – original draft; writing – review and editing.

## CONFLICT OF INTEREST

All authors report no conflict of interest.

## Supporting information


Appendix A–K

## Data Availability

The data that support the findings of this study are openly available in “figshare” at https://figshare.com/s/d7194f6f2f18082e623c. One exemplary test scene of the BASIT‐ToM at three intensity levels of the portrayed paralinguistic cues can be found in “figshare” at https://figshare.com/s/5ca4ff99790fcc5866db.

## References

[jnp12290-bib-0001] Abu‐Akel, A. , & Bailey, A. L. (2000). Correspondence. Psychological Medicine, 30(3), 735–738. 10883728 10.1017/s0033291799002123

[jnp12290-bib-0002] American Psychiatric Association . (2013). Diagnostic and statistical manual of mental disorders: DSM‐5 (Vol. 5). American Psychiatric Association.

[jnp12290-bib-0003] Amodio, D. M. , & Frith, C. D. (2006). Meeting of minds: The medial frontal cortex and social cognition. Nature Reviews Neuroscience, 7(4), 268–277. 16552413 10.1038/nrn1884

[jnp12290-bib-0004] Appelbaum, M. , Cooper, H. , Kline, R. B. , Mayo‐Wilson, E. , Nezu, A. M. , & Rao, S. M. (2018). Journal article reporting standards for quantitative research in psychology: The APA publications and communications board task force report. American Psychologist, 73(1), 3–25. 29345484 10.1037/amp0000191

[jnp12290-bib-0005] Beck, A. T. , Ward, C. H. , Mendelson, M. , Mock, J. , & Erbaugh, J. (1961). An inventory for measuring depression. Archives of General Psychiatry, 4(6), 561–571. 13688369 10.1001/archpsyc.1961.01710120031004

[jnp12290-bib-0006] Bora, E. , Walterfang, M. , & Velakoulis, D. (2015). Theory of mind in behavioural‐variant frontotemporal dementia and Alzheimer's disease: A meta‐analysis. Journal of Neurology, Neurosurgery & Psychiatry, 86(7), 714–719. 25595152 10.1136/jnnp-2014-309445

[jnp12290-bib-0007] Canty, A. L. , Neumann, D. L. , Fleming, J. , & Shum, D. H. (2015). Evaluation of a newly developed measure of theory of mind: The virtual assessment of mentalising ability. Neuropsychological Rehabilitation, 27(5), 834–870. 26095322 10.1080/09602011.2015.1052820

[jnp12290-bib-0008] Canty, A. L. , Neumann, D. L. , & Shum, D. H. (2017). Using virtual reality to assess theory of mind subprocesses and error types in early and chronic schizophrenia. Schizophrenia Research: Cognition, 10, 15–19. 29114452 10.1016/j.scog.2017.09.001 PMC5622995

[jnp12290-bib-0009] Castelli, I. , Pini, A. , Alberoni, M. , Liverta‐Sempio, O. , Baglio, F. , Massaro, D. , Marchetti, A. , & Nemni, R. (2011). Mapping levels of theory of mind in Alzheimer's disease: A preliminary study. Aging & Mental Health, 15(2), 157–168. 21140304 10.1080/13607863.2010.513038

[jnp12290-bib-0010] Corradi‐Dell'Acqua, C. , Ronchi, R. , Thomasson, M. , Bernati, T. , Saj, A. , & Vuilleumier, P. (2020). Deficits in cognitive and affective theory of mind relate to dissociated lesion patterns in prefrontal and insular cortex. Cortex, 128, 218–233. 32380282 10.1016/j.cortex.2020.03.019

[jnp12290-bib-0011] Cotter, J. , Granger, K. , Backx, R. , Hobbs, M. , Looi, C. Y. , & Barnett, J. H. (2018). Social cognitive dysfunction as a clinical marker: A systematic review of meta‐analyses across 30 clinical conditions. Neuroscience & Biobehavioral Reviews, 84, 92–99. 29175518 10.1016/j.neubiorev.2017.11.014

[jnp12290-bib-0012] Dziobek, I. , Fleck, S. , Kalbe, E. , Rogers, K. , Hassenstab, J. , Brand, M. , Kessler, J. , Woike, J. K. , Wolf, O. T. , & Convit, A. (2006). Introducing MASC: A movie for the assessment of social cognition. Journal of Autism and Developmental Disorders, 36(5), 623–636. 16755332 10.1007/s10803-006-0107-0

[jnp12290-bib-0013] Fortier, J. , Besnard, J. , & Allain, P. (2018). Theory of mind, empathy and emotion perception in cortical and subcortical neurodegenerative diseases. Revue Neurologique, 174(4), 237–246. 29622366 10.1016/j.neurol.2017.07.013

[jnp12290-bib-0014] Frith, C. D. (2004). Schizophrenia and theory of mind. Psychological Medicine, 34(3), 385–389. 15259823 10.1017/s0033291703001326

[jnp12290-bib-0015] Genova, H. M. , & McDonald, S. (2020). Social cognition in individuals with progressive multiple sclerosis: A pilot study using TASIT‐S. Journal of the International Neuropsychological Society, 26(5), 539–544. 31948498 10.1017/S1355617719001371

[jnp12290-bib-0016] Ghosh, B. C. , Calder, A. J. , Peers, P. V. , Lawrence, A. D. , Acosta‐Cabronero, J. , Pereira, J. M. , Hodges, J. R. , & Rowe, J. B. (2012). Social cognitive deficits and their neural correlates in progressive supranuclear palsy. Brain, 135(7), 2089–2102. 22637582 10.1093/brain/aws128 PMC3381722

[jnp12290-bib-0017] Henry, J. D. , Phillips, L. H. , & Von Hippel, C. (2014). A meta‐analytic review of theory of mind difficulties in behavioural‐variant frontotemporal dementia. Neuropsychologia, 56, 53–62. 24412701 10.1016/j.neuropsychologia.2013.12.024

[jnp12290-bib-0018] Henry, J. D. , Von Hippel, W. , Molenberghs, P. , Lee, T. , & Sachdev, P. S. (2016). Clinical assessment of social cognitive function in neurological disorders. Nature Reviews Neurology, 12(1), 28–39. 26670297 10.1038/nrneurol.2015.229

[jnp12290-bib-0019] Honan, C. A. , McDonald, S. , Sufani, C. , Hine, D. W. , & Kumfor, F. (2016). The awareness of social inference test: Development of a shortened version for use in adults with acquired brain injury. The Clinical Neuropsychologist, 30(2), 243–264. 10.1080/13854046.2015.1136691 26918817

[jnp12290-bib-0060] Jarsch, M. , Bartsch, K. , Berres, M. , Ryf, I. , Jurisic, K. , Sluka, C. , Kressig, R. W. , Monsch, A. U. , McDonald, S. , Piguet, O. , & Sollberger, M. (2022). The Basel Version of the Awareness of Social Inference Test‐Emotion Recognition (BASIT‐ER): Preliminary validation analyses in healthy adults. Neuropsychology, 36(2), 175–184. 10.1037/neu0000786 34928635

[jnp12290-bib-0020] Kennedy, D. P. , & Adolphs, R. (2012). The social brain in psychiatric and neurological disorders. Trends in Cognitive Sciences, 16(11), 559–572. 10.1016/j.tics.2012.09.006 23047070 PMC3606817

[jnp12290-bib-0021] Kumfor, F. , Honan, C. , McDonald, S. , Hazelton, J. L. , Hodges, J. R. , & Piguet, O. (2017). Assessing the “social brain” in dementia: Applying TASIT‐S. Cortex, 93, 166–177. 28662418 10.1016/j.cortex.2017.05.022

[jnp12290-bib-0022] Lancaster, K. , Stone, E. M. , & Genova, H. M. (2019). Cognitive but not affective theory of mind deficits in progressive MS. Journal of the International Neuropsychological Society, 25(8), 896–900. 31196250 10.1017/S1355617719000584

[jnp12290-bib-0023] Lavoie, M. A. , Vistoli, D. , Sutliff, S. , Jackson, P. L. , & Achim, A. M. (2016). Social representations and contextual adjustments as two distinct components of the theory of mind brain network: Evidence from the REMICS task. Cortex, 81, 176–191. 10.1016/j.cortex.2016.04.017 27236373

[jnp12290-bib-0024] Lewis, P. A. , Birch, A. , Hall, A. , & Dunbar, R. I. M. (2017). Higher order intentionality tasks are cognitively more demanding. Social Cognitive and Affective Neuroscience, 12(7), 1063–1071. 10.1093/scan/nsx034 28338962 PMC5490680

[jnp12290-bib-0025] Martinez, G. , Alexandre, C. , Mam‐Lam‐Fook, C. , Bendjemaa, N. , Gaillard, R. , Garel, P. , Dziobek, I. , Amado, I. , & Krebs, M. O. (2017). Phenotypic continuum between autism and schizophrenia: Evidence from the movie for the assessment of social cognition (MASC). Schizophrenia Research, 185, 161–166. 10.1016/j.schres.2017.01.012 28089135

[jnp12290-bib-0029] McDonald, S. (2012). New frontiers in neuropsychological assessment: Assessing social perception using a standardised instrument, the awareness of social inference test. Australian Psychologist, 47(1), 39–48.

[jnp12290-bib-0030] McDonald, S. , Bornhofen, C. , Shum, D. , Long, E. , Saunders, C. , & Neulinger, K. (2006). Reliability and validity of the awareness of social inference test (TASIT): A clinical test of social perception. Disability and Rehabilitation, 28(24), 1529–1542. 17178616 10.1080/09638280600646185

[jnp12290-bib-0031] McDonald, S. , Fisher, A. , Flanagan, S. , & Honan, C. A. (2017). Impaired perception of sincerity after severe traumatic brain injury. Journal of Neuropsychology, 11(2), 291–304. 26482440 10.1111/jnp.12086

[jnp12290-bib-0032] McDonald, S. , Fisher, A. , Togher, L. , Tate, R. , Rushby, J. , English, T. , Kelly, M. , Mathersul, D. , Froreich, F. , & Francis, H. (2015). Adolescent performance on the awareness of social inference test: TASIT. Brain Impairment, 16(1), 3–18.

[jnp12290-bib-0033] McDonald, S. , Flanagan, S. , Martin, I. , & Saunders, C. (2004). The ecological validity of TASIT: A test of social perception. Neuropsychological Rehabilitation, 14(3), 285–302.

[jnp12290-bib-0034] McDonald, S. , Flanagan, S. , Rollins, J. , & Kinch, J. (2003). TASIT: A new clinical tool for assessing social perception after traumatic brain injury. The Journal of Head Trauma Rehabilitation, 18(3), 219–238. 12802165 10.1097/00001199-200305000-00001

[jnp12290-bib-0035] Meijering, B. , Van Rijn, H. , Taatgen, N. , & Verbrugge, R. (2011). I do know what you think I think: Second‐order theory of mind in strategic games is not that difficult . Paper presented at the Proceedings of the Annual Meeting of the Cognitive Science Society.

[jnp12290-bib-0036] Montag, C. , Ehrlich, A. , Neuhaus, K. , Dziobek, I. , Heekeren, H. R. , Heinz, A. , & Gallinat, J. (2010). Theory of mind impairments in euthymic bipolar patients. Journal of Affective Disorders, 123(1–3), 264–269. 10.1016/j.jad.2009.08.017 19748680

[jnp12290-bib-0037] Nasreddine, Z. S. , Phillips, N. A. , Bédirian, V. , Charbonneau, S. , Whitehead, V. , Collin, I. , Cummings, J. L. , & Chertkow, H. (2005). The Montreal cognitive assessment, MoCA: A brief screening tool for mild cognitive impairment. Journal of the American Geriatrics Society, 53(4), 695–699. 15817019 10.1111/j.1532-5415.2005.53221.x

[jnp12290-bib-0038] Nosek, B. , Alter, G. , & Banks, G. (2015). SCIENTIFIC STANDARDS. Promoting an open research culture. Science, 348(6242), 1422–1425. 26113702 10.1126/science.aab2374 PMC4550299

[jnp12290-bib-0039] Peirce, J. W. (2007). PsychoPy—Psychophysics software in python. Journal of Neuroscience Methods, 162(1–2), 8–13. 17254636 10.1016/j.jneumeth.2006.11.017 PMC2018741

[jnp12290-bib-0040] Peirce, J. W. (2009). Generating stimuli for neuroscience using PsychoPy. Frontiers in Neuroinformatics, 2, 10. 19198666 10.3389/neuro.11.010.2008 PMC2636899

[jnp12290-bib-0041] Poletti, M. , Enrici, I. , & Adenzato, M. (2012). Cognitive and affective theory of mind in neurodegenerative diseases: Neuropsychological, neuroanatomical and neurochemical levels. Neuroscience & Biobehavioral Reviews, 36(9), 2147–2164. 22819986 10.1016/j.neubiorev.2012.07.004

[jnp12290-bib-0042] Pottgen, J. , Dziobek, I. , Reh, S. , Heesen, C. , & Gold, S. M. (2013). Impaired social cognition in multiple sclerosis. Journal of Neurology, Neurosurgery, and Psychiatry, 84(5), 523–528. 10.1136/jnnp-2012-304157 23315621

[jnp12290-bib-0043] Quidé, Y. , Wilhelmi, C. , & Green, M. J. (2020). Structural brain morphometry associated with theory of mind in bipolar disorder and schizophrenia. PsyCh Journal, 9(2), 234–246. 31773921 10.1002/pchj.322

[jnp12290-bib-0044] Quinting, J. , Jonas, K. , Wendt, C. , & Stenneken, P. (2020). Social cognition in cognitive‐communication disorders following traumatic brain injury: A video‐based assessment. Aphasie Und Verwandte Gebiete, 48(2), 6–18.

[jnp12290-bib-0045] R Core Team . (2020). R: A language and environment for statistical computing. R Foundation for Statistical Computing.

[jnp12290-bib-0046] R Studio Team . (2016). RStudio: Integrated development environment for R (1.1.463). RStudio, Inc.

[jnp12290-bib-0047] Rankin, K. P. , Salazar, A. , Gorno‐Tempini, M. L. , Sollberger, M. , Wilson, S. M. , Pavlic, D. , Stanley, C. M. , Glenn, S. , Weiner, M. W. , & Miller, B. L. (2009). Detecting sarcasm from paralinguistic cues: Anatomic and cognitive correlates in neurodegenerative disease. NeuroImage, 47(4), 2005–2015. 19501175 10.1016/j.neuroimage.2009.05.077 PMC2720152

[jnp12290-bib-0048] Rasch, G. (1960). Probabilistic models for some intelligence and attainment tests. Paedagogike Institut.

[jnp12290-bib-0049] Rascovsky, K. , Hodges, J. R. , Knopman, D. , Mendez, M. F. , Kramer, J. H. , Neuhaus, J. , van Swieten, J. C. , Seelaar, H. , Dopper, E. G. , Onyike, C. U. , Hillis, A. E. , Josephs, K. A. , Boeve, B. F. , Kertesz, A. , Seeley, W. W. , Rankin, K. P. , Johnson, J. K. , Gorno‐Tempini, M. L. , Rosen, H. , … Miller, B. L. (2011). Sensitivity of revised diagnostic criteria for the behavioural variant of frontotemporal dementia. Brain, 134(9), 2456–2477. 21810890 10.1093/brain/awr179 PMC3170532

[jnp12290-bib-0050] Rizopoulos, D. (2006). Ltm: An R package for latent variable modeling and item response analysis. Journal of Statistical Software, 17, 1–25.

[jnp12290-bib-0051] Rossetto, F. , Castelli, I. , Baglio, F. , Massaro, D. , Alberoni, M. , Nemni, R. , Shamay‐Tsoory, S. , & Marchetti, A. (2018). Cognitive and affective theory of mind in mild cognitive impairment and Parkinson's disease: Preliminary evidence from the italian version of the yoni task. Developmental Neuropsychology, 43(8), 764–780. 30299987 10.1080/87565641.2018.1529175

[jnp12290-bib-0052] Ryan, N. P. , Catroppa, C. , Beare, R. , Silk, T. J. , Hearps, S. J. , Beauchamp, M. H. , Yeates, K. O. , & Anderson, V. A. (2017). Uncovering the neuroanatomical correlates of cognitive, affective and conative theory of mind in paediatric traumatic brain injury: A neural systems perspective. Social Cognitive and Affective Neuroscience, 12(9), 1414–1427. 28505355 10.1093/scan/nsx066 PMC5629820

[jnp12290-bib-0053] Sharp, C. , Pane, H. , Ha, C. , Venta, A. , Patel, A. B. , Sturek, J. , & Fonagy, P. (2011). Theory of mind and emotion regulation difficulties in adolescents with borderline traits. Journal of the American Academy of Child and Adolescent Psychiatry, 50(6), 563–573 e561. 10.1016/j.jaac.2011.01.017 21621140

[jnp12290-bib-0054] Thomann, A. E. , Goettel, N. , Monsch, R. J. , Berres, M. , Jahn, T. , Steiner, L. A. , & Monsch, A. U. (2018). The Montreal cognitive assessment: Normative data from a German‐speaking cohort and comparison with international normative samples. Journal of Alzheimer's Disease, 64(2), 643–655. 10.3233/JAD-180080 PMC6027948 29945351

[jnp12290-bib-0055] Wang, Y. Y. , Wang, Y. , Zou, Y. M. , Ni, K. , Tian, X. , Sun, H. W. , Lui, S. S. Y. , Cheung, E. F. C. , Suckling, J. , & Chan, R. C. K. (2018). Theory of mind impairment and its clinical correlates in patients with schizophrenia, major depressive disorder and bipolar disorder. Schizophrenia Research, 197, 349–356. 29122405 10.1016/j.schres.2017.11.003

[jnp12290-bib-0056] Westerhof‐Evers, H. J. , Visser‐Keizer, A. C. , McDonald, S. , & Spikman, J. M. (2014). Performance of healthy subjects on an ecologically valid test for social cognition: The short, Dutch version of the awareness of social inference test (TASIT). Journal of Clinical and Experimental Neuropsychology, 36(10), 1031–1041. 25380130 10.1080/13803395.2014.966661

[jnp12290-bib-0057] Yesavage, J. A. , & Sheikh, J. I. (1986). 9/geriatric depression scale (GDS) recent evidence and development of a shorter version. Clinical Gerontologist, 5(1–2), 165–173.

