# Supplementary material for: Development of the Basel Version of the Awareness of Social Inference Test – Theory of Mind (BASIT‐ToM) in healthy adults
Source: J Neuropsychol. 2022 Sep 21;17(1):125–45. doi: 10.1111/jnp.12290 (PMC10947008; doi:10.1111/jnp.12290)
Supplement: Supplementary file 1 — Appendix A–K [file JNP-17-125-s001.docx]

**Supplemental Material**

Development of the Basel Version of the Awareness of Social Inference Test – Theory of Mind (BASIT-ToM) in Healthy Adults

Table of Contents

[Appendix A 6](#_Toc86057356)

[Table A1 6](#_Toc86057357)

[Evaluation Criteria of the TASIT-SIM Scenes for Scene Selection 6](#_Toc86057358)

[Appendix B 7](#_Toc86057359)

[Table B1.1 7](#_Toc86057360)

[Evaluation and Selection of the Honesty Scenes from TASIT-SIM Form A and Form B 7](#_Toc86057361)

[Table B1.2 9](#_Toc86057362)

[Evaluation and Selection of the Simple Sarcasm Scenes from TASIT-SIM Form A and Form B 9](#_Toc86057363)

[Table B1.3 11](#_Toc86057364)

[Evaluation and Selection of the Paradoxical Sarcasm Scenes from TASIT-SIM Form A and Form B 11](#_Toc86057365)

[Table B2 13](#_Toc86057366)

[Selected Scenes for the BASIT-ToM Film Project 13](#_Toc86057367)

[Appendix C 14](#_Toc86057368)

[Document C1 14](#_Toc86057369)

[Screenplays of the BASIT-ToM Scenes 14](#_Toc86057370)

[Table C1 28](#_Toc86057371)

[Main Changes of the Script (TASIT-SIM → BASIT-ToM)](#_Toc86057372) 28

[Appendix D 29](#_Toc86057373)

[Table D1 29](#_Toc86057374)

[Distribution of the Actors to the BASIT-ToM and Corresponding TASIT-SIM Scenes 29](#_Toc86057375)

[Appendix E 30](#_Toc86057376)

[Document E1 30](#_Toc86057377)

[Background Information of each BASIT-ToM Intensity Version Scene for the Actors 30](#_Toc86057378)

[Appendix F 47](#_Toc86057379)

[Table F1: Scene H#1 47](#_Toc86057380)

[Test Questions of the BASIT-ToM and TASIT-SIM with their Corresponding Correct Answers, Types and Orders of Theory of Mind 47](#_Toc86057381)

[Table F2: Scene H#2 49](#_Toc86057382)

[Test Questions of the BASIT-ToM and TASIT-SIM with their Corresponding Correct Answers, Types and Orders of Theory of Mind 49](#_Toc86057383)

[Table F3: Scene H#3 51](#_Toc86057384)

[Test Questions of the BASIT-ToM and TASIT-SIM with their Corresponding Correct Answers, Types and Orders of Theory of Mind 51](#_Toc86057385)

[Table F4: Scene H#4 53](#_Toc86057386)

[Test Questions of the BASIT-ToM and TASIT-SIM with their Corresponding Correct Answers, Types and Orders of Theory of Mind 53](#_Toc86057387)

[Table F5: Scene sS#1 55](#_Toc86057388)

[Test Questions of the BASIT-ToM and TASIT-SIM with their Corresponding Correct Answers, Types and Orders of Theory of Mind 55](#_Toc86057389)

[Table F6: Scene sS#2 57](#_Toc86057390)

[Test Questions of the BASIT-ToM and TASIT-SIM with their Corresponding Correct Answers, Types and Orders of Theory of Mind 57](#_Toc86057391)

[Table F7: Scene sS#3 59](#_Toc86057392)

[Test Questions of the BASIT-ToM and TASIT-SIM with their Corresponding Correct Answers, Types and Orders of Theory of Mind 59](#_Toc86057393)

[Table F8: Scene sS#4 61](#_Toc86057394)

[Test Questions of the BASIT-ToM and TASIT-SIM with their Corresponding Correct Answers, Types and Orders of Theory of Mind 61](#_Toc86057395)

[Table F9: Scene pS#1 63](#_Toc86057396)

[Test Questions of the BASIT-ToM and TASIT-SIM with their Corresponding Correct Answers, Types and Orders of Theory of Mind 63](#_Toc86057397)

[Table F10: Scene pS#2 65](#_Toc86057398)

[Test Questions of the BASIT-ToM and TASIT-SIM with their Corresponding Correct Answers, Types and Orders of Theory of Mind 65](#_Toc86057399)

[Table F11: Scene pS#3 67](#_Toc86057400)

[Test Questions of the BASIT-ToM and TASIT-SIM with their Corresponding Correct Answers, Types and Orders of Theory of Mind 67](#_Toc86057401)

[Table F12: Scene pS#4 69](#_Toc86057402)

[Test Questions of the BASIT-ToM and TASIT-SIM with their Corresponding Correct Answers, Types and Orders of Theory of Mind 69](#_Toc86057403)

[Table F13: Scene Practice(pS) 71](#_Toc86057402)

[Test Questions of the BASIT-ToM and TASIT-SIM with their Corresponding Correct Answers, Types and Orders of Theory of Mind 71](#_Toc86057403)

[Appendix G 73](#_Toc86057404)

[Table G1 73](#_Toc86057405)

Main [Differences between the BASIT-ToM and TASIT-SIM 73](#_Toc86057406)

[Appendix H 75](#_Toc86057407)

[Document H1 75](#_Toc86057408)

[Details of the Programming and Data Storage 75](#_Toc86057409)

[Appendix I 79](#_Toc86057410)

[Figure I1 79](#_Toc86057411)

[Exemplary Representation of the BASIT-ToM Computer-Based Application Process 79](#_Toc86057412)

[Appendix J 80](#_Toc86057413)

[Table J.1: 80](#_Toc86057414)

[Item Characteristic Curves of the Intensity Version Scenes for the Message Type Honesty 80](#_Toc86057415)

[Table J.2: 86](#_Toc86057416)

[Item Characteristic Curves of the Intensity Version Scenes for the Message Type Paradoxical Sarcasm 86](#_Toc86057417)

[Table J.3: 93](#_Toc86057416)

[Item Characteristic Curves of the Intensity Version Scenes for the Message Type Simple Sarcasm 93](#_Toc86057417)

[Appendix K 102](#_Toc86057413)

[Table K.1: 102](#_Toc86057414)

**Appendix A**

## Table A1

### Evaluation criteria of the TASIT-SIM scenes for scene selection

|  |
| --- |
| 1. The topic of the film scene is suitable for the age groups over 60 2. The topic of the film scene is suitable for people from the German-speaking cultural area |
| 1. The film scene appears realistic and comprehensible |
| 1. The content of the film scene is contemporary 2. Set design appears realistic |
| 1. The message type portrayed appears appropriate for the story of the scene 2. The message type is portrayed by different modalities (e.g. gestures, facial expressions, linguistic characteristics) 3. Office or living room setting is possible 4. A change of location is not necessary during the scene 5. Gender ratio in the cast is unimportant 6. Number of actors 7. Duration of the scene is not too short |

*Note.* TASIT - SIM = The Awareness of Social Inference Test - Social Inference Minimal.

# Appendix B

## Table B1.1

### Evaluation and Selection of the Honesty Scenes from TASIT-SIM Form A and Form B

|  | Scene ID | | | | | | | | | |
| --- | --- | --- | --- | --- | --- | --- | --- | --- | --- | --- |
| Evaluation criteria | A1 | A4 | A7 | **A11** | A14 | **B1** | B4 | **B7** | **B11** | B14 |
| The topic of the film scene is suitable for the age groups over 60 | 1 | 1 | 1 | 1 | 1 | 1 | 1 | 1 | 1 | 0 |
| The topic of the film scene is suitable for people from German- speaking cultural area | 1 | 1 | 1 | 1 | 1 | 1 | 1 | 1 | 1 | 1 |
| The film scene appears realistic and comprehensible | 1 | 1 | 1 | 1 | 1 | 0 | 1 | 1 | 1 | 1 |
| The content of the film scene is contemporary | 1 | 1 | 1 | 1 | 1 | 1 | 1 | 1 | 1 | 1 |
| Set design appears realistic | 1 | 1 | 1 | 1 | 0 | 1 | 1 | 1 | 0 | 0 |
| The message type portrayed appears appropriate for the story of the scene | 0 | 0 | 0 | 1 | 0 | 1 | 0 | 1 | 1 | 1 |
| The message type is portrayed by different modalities (e.g. gestures, facial expressions, linguistic characteristics) | 1 | 1 | 1 | 1 | 1 | 1 | 1 | 1 | 1 | 1 |
| Office or living room setting is possible | 1 | 1 | 1 | 1 | 1 | 1 | 1 | 1 | 1 | 1 |
|  | Scene ID | | | | | | | | | |
| Evaluation criteria | A1 | A4 | A7 | **A11** | A14 | **B1** | B4 | **B7** | **B11** | B14 |
| A change of location is not necessary during the scene | 1 | 1 | 1 | 1 | 1 | 1 | 1 | 1 | 1 | 1 |
| Gender ratio in the cast is unimportant^†^ | 1 | 1 | 1 | 1 | 1 | 1 | 1 | 1 | 1 | 1 |
| Number of actors^†^ | 2 | 1 | 2 | 2 | 2 | 2 | 2 | 2 | 2 | 2 |
| Duration of the scene is not too short^†^ | 1 | 1 | 1 | 1 | 1 | 1 | 1 | 1 | 1 | 1 |
| **Total score** | 8/9 | 8/9 | 8/9 | 9/9 | 7/9 | 8/9 | 8/9 | 9/9 | 8/9 | 7/9 |

*Note.* TASIT-SIM = The Awareness of Social Inference Test – Social Inference Minimal; A = TASIT-SIM form A,

B = TASIT-SIM form B. Scoring: 0 = criterion was not fulfilled; 1 = criterion was fulfilled. Selected scenes are in bold. ^†^Criterion is not part of the total score.

## Table B1.2

*Evaluation and Selection of the Simple Sarcasm Scenes from TASIT-SIM Form A and*

*Form B*

|  | Scene ID | | | | | | | | | |
| --- | --- | --- | --- | --- | --- | --- | --- | --- | --- | --- |
| Evaluation criteria | A2 | **A6** | A9 | A10 | A13 | **B2** | B6 | B9 | **B10** | **B13** |
| The topic of the film scene is suitable for the age groups over 60 | 1 | 1 | 1 | 1 | 1 | 1 | 1 | 1 | 1 | 1 |
| The topic of the film scene is suitable for people from German- speaking cultural area | 1 | 1 | 1 | 1 | 1 | 1 | 1 | 1 | 1 | 1 |
| The film scene appears realistic and comprehensible | 1 | 1 | 0 | 1 | 1 | 1 | 1 | 1 | 1 | 1 |
| The content of the film scene is contemporary | 1 | 1 | 1 | 1 | 1 | 1 | 1 | 1 | 1 | 1 |
| Set design appears realistic | 1 | 1 | 1 | 0 | 1 | 1 | 0 | 1 | 1 | 1 |
| The message type portrayed appears appropriate for the story of the scene | 0 | 1 | 0 | 0 | 1 | 1 | 1 | 0 | 1 | 0 |
| The message type is portrayed by different modalities (e.g. gestures, facial expressions, linguistic characteristics) | 1 | 1 | 1 | 1 | 1 | 1 | 1 | 1 | 1 | 1 |
| Office or living room setting is possible | 1 | 1 | 1 | 1 | 1 | 1 | 1 | 1 | 1 | 1 |
|  | Scene ID | | | | | | | | | |
| Evaluation criteria | A2 | **A6** | A9 | A10 | A13 | **B2** | B6 | B9 | **B10** | **B13** |
| A change of location is not necessary during the scene | 1 | 1 | 1 | 1 | 1 | 1 | 1 | 1 | 1 | 1 |
| Gender ratio in the cast is unimportant^†^ | 1 | 1 | 1 | 1 | 1 | 1 | 1 | 1 | 0 | 0 |
| Number of actors^†^ | 2 | 2 | 2 | 2 | 2 | 2 | 1 | 2 | 2 | 2 |
| Duration of the scene is not too short^†^ | 1 | 1 | 1 | 1 | 1 | 1 | 1 | 1 | 1 | 1 |
| **Total score** | 8/9 | 8/9 | 8/9 | 9/9 | 7/9 | 8/9 | 8/9 | 9/9 | 8/9 | 7/9 |

*Note.* TASIT-SIM = The Awareness of Social Inference Test – Social Inference Minimal; A = TASIT-SIM form A,

B = TASIT-SIM form B. Scoring: 0 = criterion was not fulfilled; 1 = criterion was fulfilled. Selected scenes are in bold. ^†^Criterion is not part of the total score.

## Table B1.3

*Evaluation and Selection of the Paradoxical Sarcasm Scenes from TASIT-SIM Form A and Form B*

|  | Scene ID | | | | | | | | | |
| --- | --- | --- | --- | --- | --- | --- | --- | --- | --- | --- |
| Evaluation criteria | **A3** | A5 | A8 | A12 | **A15** | B3 | **B5** | **B8** | B12 | **B15** |
| The topic of the film scene is suitable for the age groups over 60 | 1 | 1 | 1 | 1 | 1 | 1 | 1 | 1 | 1 | 1 |
| The topic of the film scene is suitable for people from German- speaking cultural area | 1 | 1 | 1 | 1 | 1 | 1 | 1 | 1 | 1 | 1 |
| The film scene appears realistic and comprehensible | 1 | 1 | 1 | 1 | 1 | 1 | 1 | 1 | 0 | 1 |
| The content of the film scene is contemporary | 1 | 1 | 1 | 1 | 1 | 1 | 1 | 1 | 1 | 1 |
| Set design appears realistic | 1 | 1 | 0 | 1 | 1 | 0 | 1 | 1 | 0 | 1 |
| The message type portrayed appears appropriate for the story of the scene | 0 | 0 | 1 | 1 | 1 | 1 | 1 | 1 | 1 | 1 |
| The message type is portrayed by different modalities (e.g. gestures, facial expressions, linguistic characteristics) | 1 | 1 | 1 | 1 | 1 | 1 | 1 | 1 | 1 | 1 |
| Office or living room setting is possible | 1 | 1 | 1 | 1 | 1 | 1 | 1 | 1 | 1 | 1 |
|  | Scene ID | | | | | | | | | |
| Evaluation criteria | **A3** | A5 | A8 | A12 | **A15** | B3 | **B5** | **B8** | B12 | **B15** |
| A change of location is not necessary during the scene | 1 | 1 | 1 | 1 | 1 | 1 | 1 | 1 | 1 | 1 |
| Gender ratio in the cast is unimportant^†^ | 1 | 1 | 1 | 1 | 1 | 0 | 1 | 0 | 1 | 1 |
| Number of actors^†^ | 2 | 2 | 2 | 2 | 2 | 2 | 2 | 2 | 2 | 2 |
| Duration of the scene is not too short^†^ | 1 | 0 | 1 | 0 | 1 | 0 | 1 | 1 | 1 | 1 |
| **Total score** | 8/9 | 8/9 | 8/9 | 9/9 | 7/9 | 8/9 | 8/9 | 9/9 | 8/9 | 7/9 |

*Note.* TASIT-SIM = The Awareness of Social Inference Test – Social Inference Minimal; A = TASIT-SIM form A,

B = TASIT-SIM form B. Scoring: 0 = criterion was not fulfilled; 1 = criterion was fulfilled. Selected scenes are in bold. ^†^Criterion is not part of the total score.

|  |  |  |  |  |  |  |  |  |  |  |
| --- | --- | --- | --- | --- | --- | --- | --- | --- | --- | --- |

## Table B2

### Selected Scenes for the BASIT-ToM Film Project

| Message type | Scene 1 | Scene 2 | Scene 3 | Scene 4 | Scene 5 |
| --- | --- | --- | --- | --- | --- |
| Honesty | B11 | A11 | B1 | B7 |  |
| Simple Sarcasm | A6 | B2 | B10 | B13 |  |
| Paradoxical Sarcasm | A3 | A15 | B5 | B8 | B15 |

*Note*. BASIT-ToM = Basel Version of the Awareness of Social Inference Test – Theory of Mind. The scene code consists of a letter indicating the form of The Awareness of Social Inference Test – Social Inference Minimal (i.e. form A or form B) and a number indicating the respective scene number.

# Appendix C

## Document C1

### Screenplays of the BASIT-ToM Scenes

**

**

**

**

**

**

**

**

## Table C1

### Main Changes of the Script (TASIT-SIM → BASIT-ToM)

| Scene | Text removed |  | Text added |
| --- | --- | --- | --- |
| H#1 (B11) | Actor repeats what another actor says several times in TASIT-SIM. These repetitions have been deleted.  M: " I feel as if I can see it another thousand times” |  | The deleted repetitions were replaced by "oh yeah". |
| H#2 (A11) |  |  | F: "I need another signature here." |
| H#3 (B1) | F: “…in between making coffee and answering telephone calls” |  |  |
| H#4 (B7) | F: “…you’re indispensable” |  |  |
| Practice scene (B15) |  |  | F: “…and these window sills!” |

*Note.* TASIT-SIM = The Awareness of Social Inference Test – Social Inference Minimal; BASIT-ToM = Basel Version of the Awareness of Social Inference Test – Theory of Mind. Scene code consists of a BASIT-ToM scene number (scene) and a letter (message type, H = Honesty). Scene code in the brackets denotes a TASIT-SIM scene consisting of form A or form B and a scene number. M = male actor, F = female actor.

# Appendix D

## Table D1

### Distribution of the Actors to the BASIT-ToM and Corresponding TASIT-SIM Scenes

| Scene | BASIT-ToM | TASIT-SIM |
| --- | --- | --- |
| Honesty |  |  |
| B11 | **M1**+F1 | **M**+F |
| A11 | **M2**+F2 | **M**+M |
| B1 | **F3**+M3 | M+**F** |
| B7 | **F4**+M4 | M+**F** |
| Simple Sarcasm |  |  |
| A6 | **F1**+M3 | M+**F** |
| B2 | **M1**+F3 | **M**+F |
| B10 | **M2**+F4 | **M**+F |
| B13 | **F2**+M4 | M+**F** |
| Paradoxical Sarcasm |  |  |
| A3 | **F3**+M2 | M+**F** |
| A15 | **M3**+F2 | **M+**F |
| B5 | **M4**+F1 | **M**+F |
| B8 | **F4**+M1 | M+**F** |
| B15 (practice scene) | **F1**+M2 | M+**F** |

*Note.* The four female actors are labelled F1 to F4 and the four male actors are labelled M1 to M4. The actors who communicate the respective message type (i.e., Honesty, Simple Sarcasm, or Paradoxical Sarcasm) are printed in bold. BASIT-ToM = Basel Version of the Awareness of Social Inference Test – Theory of Mind; TASIT-SIM = The Awareness of Social Inference Test – Social Inference Minimal.

# Appendix E

## Document E1

Background Information of each BASIT-ToM Intensity Version Scene for the Actors

**Honesty 1 (B11) – Theatre**

| Main Actor: **M1**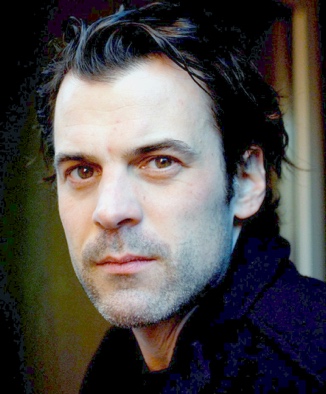 | Communication Partner: F1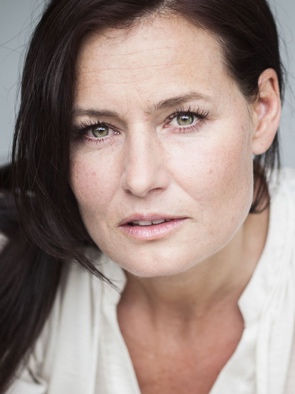 |
| --- | --- |

**Background Information**

- M1 and F1 are married. In their free time, they do a lot together and like to chat about what they have experienced together.
- M1 and F1 are teachers

**Intensities**

- **Low intensity:** M1 and F1 have seen a play the week before. M1 and F1 are sitting on the sofa. M1 is reading a book and F1 is reading a magazine that has something written about the play. They casually talk about the play. M1 concentrates on his book during the conversation.
- **Medium intensity:** M1 and F1 have seen a play the night before. They are sitting on the sofa, drinking wine and talking. They rarely go to the theatre, but found the play good.
- **High intensity:** M1 and F1 have just returned home from a visit to the theatre, are sitting on the sofa and drinking wine. Both are theatre lovers and overwhelmed by the play. They reminisce and talk animatedly.

**Honesty 2 (A11) – Tickets**

| Main Actor: **M2**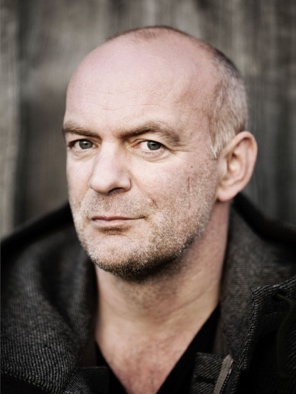 | Communication Partner: F2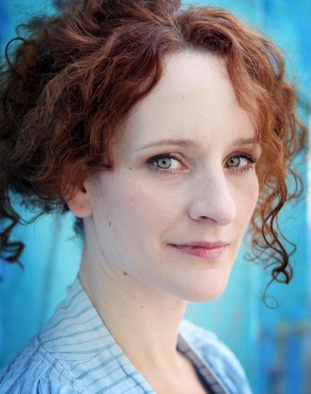 |
| --- | --- |

**Background Information**

- M2 is the boss of a company.
- F2 is M2's secretary. She supports his work in the company and takes on private organizational tasks, such as booking tickets.

**Intensities**

- **Low intensity:** F2 has organized tickets for M2 and his wife for an almost sold-out play. F2 gives him the tickets and casually clarifies to him that it was very difficult to get the tickets. He also casually thanks her while signing forms that F2 hands him.

M2 likes to watch a play from time to time, but is not a fan of it. However, he is glad to be able to make his wife happy by going to the theatre.

- **Medium intensity:** F2 has organized tickets for a gala dinner for M2 and his wife. F2 is pleased and relieved that she was able to organize seats for M2 and his wife. M2 is very much looking forward to the evening and thanks F2 for her efforts.
- **High intensity:** M2 and his wife are big Rolling Stones fans. F2 was able to organize tickets for the last Rolling Stones concert. Now, she proudly shows them to her husband. M2 feels excited anticipation and is full of gratitude that F2 organized the tickets for him.

**Honesty 3 (B1) – Hard Day**

| Main Actor: **F3**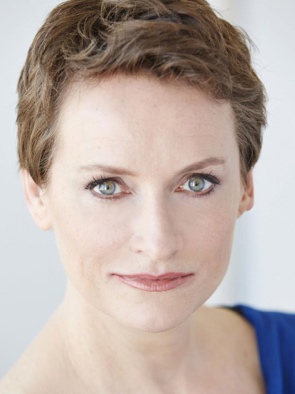 | Communication Partner: M3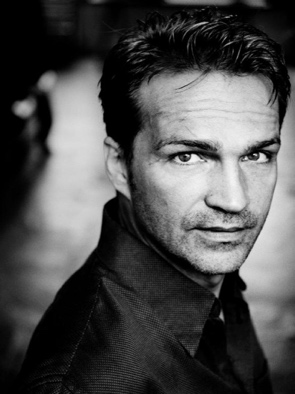 |
| --- | --- |

**Background Information**

- F3 is the head of a small law firm with few employees.
- M3 is a lawyer.

**Intensities**

- **Low intensity:** In the law firm, old files have to be sorted out and destroyed once a year. This work has to be done in addition to the daily work tasks. M3 works as a freelancer in the law office. F3 has asked him to support her in this annual cleanup. While she concentrates on sorting out the last files, M3 says goodbye. It is actually a matter of course for both of them that M3 will help again the next day. Hence, the conversation is held casually.
- **Medium intensity:** The law firm is preparing for a difficult trial that will take place in three days. M3, as a part-time worker, only works 3 days a week. F3 has asked him to come to work on extra days so they can manage the workload. F3 is very happy that M3 helps. For M3 it is a matter of course that he supports his colleagues. Nevertheless, he is happy that his efforts are appreciated.
- **High intensity:** The law firm has to move this week. The employee who should have helped organize the move has called in sick at short notice. M3 has come back from his holiday to help. F3 is very relieved that M3 is willing to do this and thanks him profusely for his help. For M3 it is a matter of course that he supports his colleagues. Nevertheless, he is pleased that his efforts are appreciated.

**Honesty 4 (B7) – Promotion**

| Main Actor: **F4**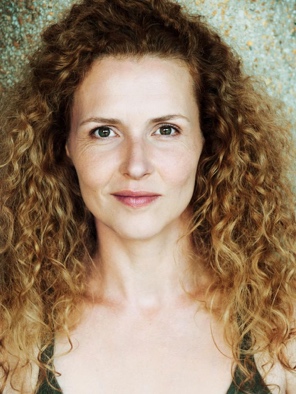 | Communication Partner: M4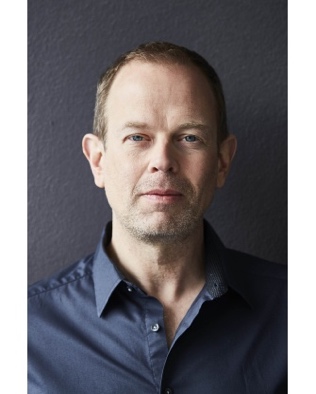 |
| --- | --- |

**Background information**

- M4 is a team leader and wants to be promoted to head of department.
- F4 is a member of M4's team.
- They work in the district office.

**Intensities**

- **Low intensity:** The conversation takes place while they are working together. Meanwhile, M4 wants to talk to F4 for a bit and therefore brings up the subject of the promotion. However, he is not very worried that it might not work out. In the previous years, the boss was always satisfied with his work. F4 encourages M4 while they both continue to concentrate on their work. The whole conversation has the character of small talk.
- **Medium intensity:** M4 and F4 are friends. M4 is worried that his boss was not satisfied with his work and someone else will be promoted. F4 wants to reassure and encourage him. They focus on the conversation and not on their work.
- **High intensity:** F4 is in love with M4. She thinks he is a great employee and a promotion is long overdue. When M4 expresses concerns about the possible promotion, she seizes the opportunity and wants to endear herself to him with her compliments.

**Simple Sarcasm 1 (A6) – “Seminar”**

| Main Actor: **F1**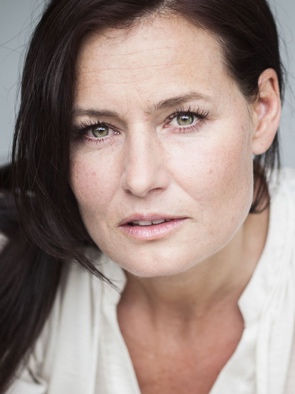 | Communication Partner: M3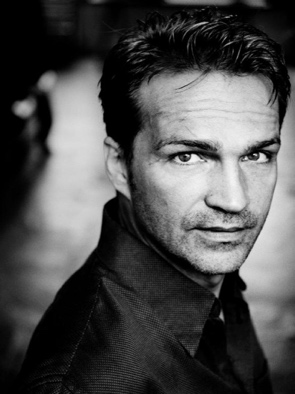 |
| --- | --- |

**Background Information**

- - F1 and M3 both work as research fellows at a university of applied sciences for business administration. In addition to their work in research, they give seminars and lectures to students and therefore have a lot to deal with professionally.
  - F1 is a single mother with two children. F1 can only reconcile work and family life with a great deal of organization and good planning. Her children often have to take a back seat and are frequently looked after by other people. As a result, F1 has a guilty conscience. She often feels stressed.
  - M3 is single, has no children and many hobbies. He is able to balance work and leisure time well. He feels neither stressed nor overwhelmed. M3 is ambitious and wants to pursue a career at the university.
  - Situation: Two weeks before the start of a seminar, F1 asked M3 to hold the seminar for her. M3 has agreed to her request. Two days before the seminar, M3 cancels.

**Intensities**

- - **Low intensity:** There was no negative history between F1 and M3. They appreciate each other as colleagues, but are not friends in their free time. M3's cancellation annoys her because she relied on his commitment. If she does not find a replacement, she will have to hold the seminar herself.
  - **Medium intensity:** F1 has the feeling that she has to perform better overall than M3. She feels that this is unfair and is therefore annoyed by M3 in advance. His cancellation makes her angry.
  - **High intensity:** M3 is known at university for not doing anything for his colleagues; he does not keep to agreements and is only concerned about his own advantage. That is why many colleagues and F1 are angry with him. F1 has promised her child to go to his school play. That is why she asked M3 to take over the seminar for her. She has explained the urgency and told him how important it is that she can rely on him. His last-minute cancellation leaves her stunned and very angry.

M3 is sincere in his apology. However, F1's problems do not affect him much. Since he wants to keep another appointment at short notice, he cancels F1 without a guilty conscience. This remains the same across all intensities.

**Simple Sarcasm 2 (B2) – Weekend away**

| Main Actor: **M1**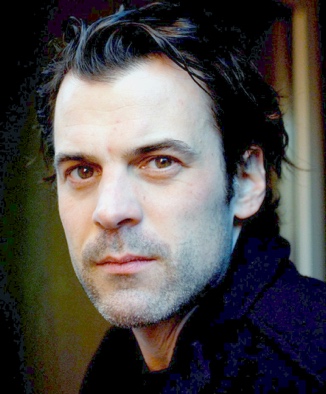 | Communication Partner: F3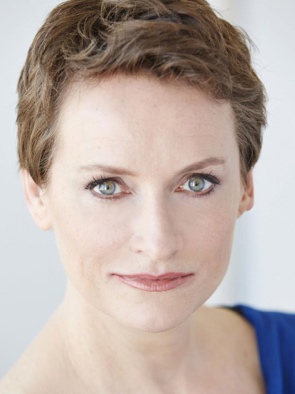 |
| --- | --- |

**Background Information**

- - F3 and M1 have been in a relationship for 10 years. They are not married and have no children. Both are very busy at work and have little free time together.
  - M1 is a café owner. He spends a lot of time in his café and mostly has to work on weekends. He is a family man. His family tries to solve all problems together. Being there for the other siblings is a high priority for him. His sister very often has minor problems for which she asks him for advice and therefore comes over. The little free time F3 and he have together is therefore often spent with his sister.
  - F3 is an estate agent. She can arrange her working hours freely, but often has to work on weekends as well. She is not a family person. She likes her sister-in-law but thinks she exaggerates her problems and comes over too often. She would like to spend more of her limited free time alone with her partner.

**Intensities**

- - **Low intensity:** It is not a special weekend outing. M1 and F3 had a small discussion beforehand about his sister's frequent appearances. However, F3 already expected M3 to ask her about his sister. Nevertheless, she is now a bit annoyed when she hears from M1 that his sister should also come along to their weekend trip.
  - **Medium intensity:** F3 and M1 had already had a small argument the day before because F3 feels that M1's sister takes up too much space in their relationship. Therefore, she is angry that his sister should now also come along to the weekend trip.
  - **High intensity:** Beforehand, there were almost daily arguments between F3 and M1 about the fact that they spend too little time together. In addition, F3 thinks that M1's sister is more important than her, which annoys her more and more and puts a lot of strain on their relationship. M1 has often promised weekend trips together and, contrary to his promises, has always invited his sister. Because F3 was very angry about this, M1 promised her that they would celebrate their ten-year anniversary as a couple on a weekend trip. Now F3 is stunned and very angry that M3 is already thinking about inviting his sister again.

M3 acts as if there has never been a discussion about his sister's frequent appearances. He is worried about his sister and therefore seriously considers taking her with them. He does not understand why F3 has a problem with this. His behaviour remains the same across all intensities.

**Simple Sarcasm 3 (B10) – Going out**

| Main Actor: **M2**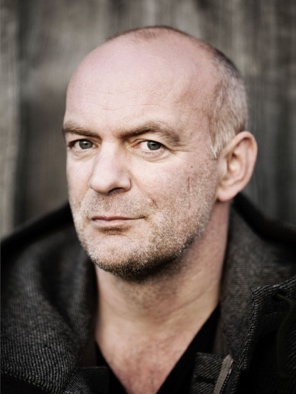 | Communication Partner: F4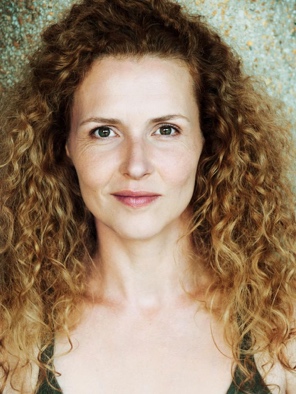 |
| --- | --- |

**Background Information**

- - M2 is an artist. He is confident, articulate and good with people.
  - F4 is a gallery owner. She is a confident, content person.

**Intensities**

- - **Low intensity:** F4 and M2 have been a happy couple for many years. They harmonize very well. Their conversations are usually full of small but affectionate banter. They like to tease each other. They do not have to give their partner any linguistic or visual clues that what they say is meant ironically. They each understand their partner's irony. Irony has become such a part of their shared conversation that they hardly notice that they are being ironic with each other.
  - **Medium intensity:** F4 and M2 have only been a couple and living together for a short time. They have a lot of fun together and flirt a lot. They often do this through an ironic communication style. They give their partner slight linguistic and visual cues (e.g. smiling) that what is said is meant ironically.
  - **High intensity:** F4 and M2 have only been a couple for a short time and have been living together for a week. They have a lot of fun together and flirt a lot. F4 came home from work late last night, when M2 was already asleep. Today she is going out alone to meet a friend for dinner; she has been doing this for a while. M2 now teases her about it, although he knows why she goes out and whom she meets. Additionally, he is also teasing her because she had already gone out yesterday and he was home alone. Quite tough in the first week of living together! Accordingly, he gives strong linguistic and visual indications that what is said is meant ironically. F4 understands M4's irony and reacts with irony.

**Simple Sarcasm 4 (B13) – Shirt**

| Main Actor: **F2**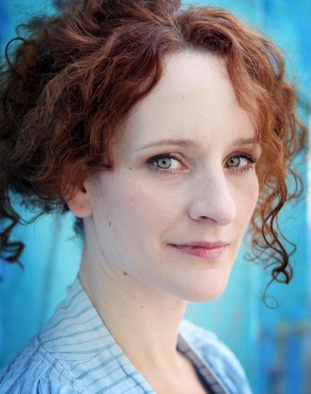 | Communication Partner: M4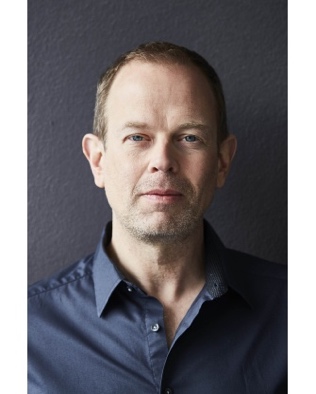 |
| --- | --- |

**Background Information**

- - F2 and M4 have been married for many years. M4 gets a shirt from his mother every year for his birthday. F2 thinks these shirts are ugly.
  - F2 is a team leader in a company.
  - M4 is a cook.
  - Situation: F2 and M4 get dressed to go to the Christmas party of F2’s company.

**Intensities**

- - **Low intensity:** F2 and M4 have no quarrel beforehand and are looking forward to the upcoming party. He tries to choose a nice shirt and wants her to like it. She has given up explaining to him, which shirts can and cannot be worn. Therefore, she "emotionlessly" to pityingly smiles at his shirt choice.
  - **Medium intensity:** F2 and M4 want to go to the Christmas party of F2’s company. At previous events, F2 has always been ashamed of her husband's poor attire. F2 has clearly told her husband several times not to wear the shirts he got from his mother. She has bought him new, fancy shirts and is annoyed when he wants to wear one of his mother's shirts again. M4 cannot understand why his wife does not like the shirts his mother regularly gives him. He wants to please his wife (F2) and therefore seriously asks her opinion.
  - **High intensity:** Immediately before the scene, F2 and M4 had a major argument. She is still angry and therefore immediately goes off the deep end when M4 also wants to wear one of his mother’s ugly shirts to her Christmas party. M4 wants to appease his wife after the argument by approaching her and asking for her opinion. In doing so, he also wants to signal to her that he is happy to go to the party.

**Paradoxical Sarcasm 1 (A3) – Report**

| Main Actor: **F3**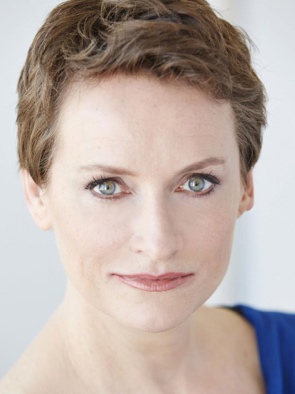 | Communication Partner: M2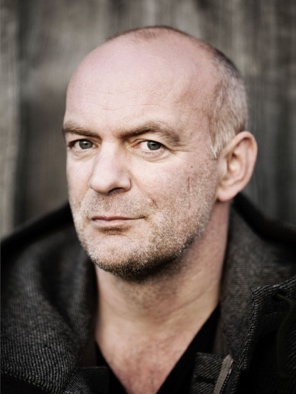 |
| --- | --- |

**Background Information**

- - F3 is a management consultant; she works efficiently and reliably and is hardworking.
  - M2 is the boss of the management consultancy. As the boss, he sets clear guidelines and has high expectations of his employees.

**Intensities**

- Situation: M2 is the boss of a management consultancy and is F3's superior. On Friday, M2 asked F3 to finish a report by Monday. On Monday morning, there is a very long report on his desk. He actually appreciates short reports, but sees the amount of work F3 put into the report over the weekend.
- **Low intensity:** M2 and F3 appreciate each other and have a collegial relationship. F3 is used to writing long reports for her boss at short notice. She has no problem with this, yet she would have liked to spend the weekend doing something else. M2 wants to convey appreciation and thanks to her by asking.
- **Medium intensity:** There is a clear hierarchical separation between M2 and F3. M2 knew that F3 hardly had time to write a report on the weekend. Nevertheless, he gave her this task because the report was urgent. Accordingly, F3 is annoyed. M2 realizes that the work for the report, given its length, was probably more time-consuming than he had imagined and now wants to convey appreciation to F3 by asking.
- **High intensity:** M2 and F3 do not have a good relationship. M2 knew that F3 did not have time to write a report at the weekend because F3 had planned to go to her mother's birthday party over the weekend. F3's mother was turning 70 and F3 had been looking forward to the party for weeks. F3 then had to write the report at night. Accordingly, she is angry with her boss.

**Paradoxical Sarcasm 2 (A15) – Earrings**

| Main Actor: **M3**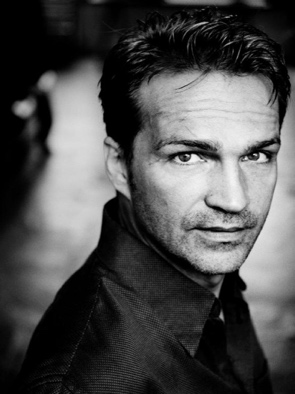 | Communication Partner:F2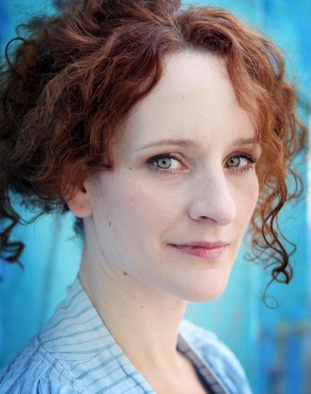 |
| --- | --- |

**Background Information**

- F2 and M3 are married and wealthy.
- M3 is an architect.
- F2 is a fashion designer.

**Intensities**

- **Low intensity:** F2 and M3 have a good relationship with F2's sister. F2 was happy to receive a gift from her. However, neither of them likes the earrings. F2's sister likes to shop at the flea market. F2 and M3 amuse themselves a little about the earrings.
- **Medium intensity:** F2 and M3 have a good relationship with F2's sister. However, she is very thrifty and has a tendency to give cheap trinkets. These earrings, which were obviously cheap, are a typical gift. Accordingly, F2 and M3 have already expected such a gift and are very amused.
- **High intensity:** For F2 and M3, expensive gifts are important. They consider cheap gifts as worthless and embarrassing. They despise F2's sister and laugh at her and her embarrassing gift.

**Paradoxical Sarcasm 3 (B5) – Meal**

| Main Actor: **M4**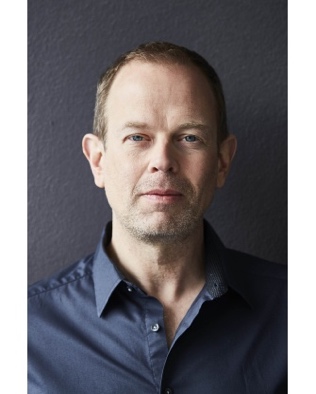 | Communication Partner: F1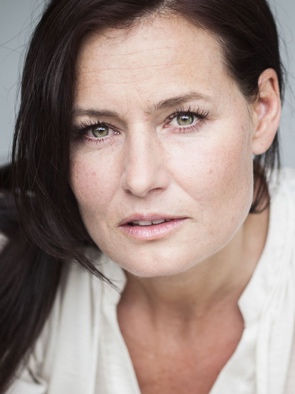 |
| --- | --- |

**Background Information**

- F1 is a journalist
- M4 is a computer scientist

**Intensities**

- **Low intensity:** F1 and M4 have been a happy couple for many years. They harmonize very well and understand each other blindly. Their conversations are usually full of small but affectionate asides. They like to tease each other. They do not have to give their partner any linguistic or visual clues that what they say is meant ironically. They each understand their partner's irony. Irony has become such a part of their shared conversation that they hardly notice that they are being ironic with each other.
- **Medium intensity:** F1 and M4 have only been a couple and living together for a short time. They have a lot of fun together and flirt a lot. They often do this through an ironic communication style. They give their partner slight linguistic and visual cues (e.g. smiling) that what is said is meant ironically.
- **High intensity:** F1 and M4 have only been a couple for a short time and have been living together for a week. They have a lot of fun together and flirt a lot. They often do this through an ironic style of communication. Now F1, who is a passionate cook, has cooked something new and is of course curious how M4 liked it. M4 knows about F3's curiosity and wants to tease her a little in the form of strong irony. He gives correspondingly strong linguistic and visual indications that what is said is meant ironically. F1 also understands M4's irony and plays along.

**Paradoxical Sarcasm 4 (B8) – Night Shift**

| Main Actor: **F4**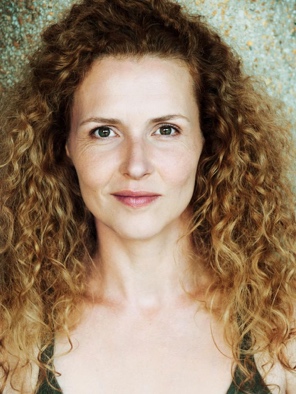 | Communication Partner: M1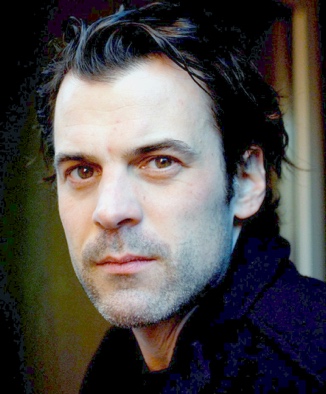 |
| --- | --- |

**Background Information**

- F4 and M1 are married.
- M1 is an air traffic controller and works in shifts He works a lot of overtime and very often fills in for other colleagues. He has very little free time.
- F4 works in a travel agency. She works normal office hours and does not have to work overtime.

**Intensities**

- **Low intensity:** It is not a special restaurant visit. Nevertheless, F4 would have liked to go out with her husband. She is a bit disappointed with M1 for not managing to turn down his boss.
- **Medium intensity:** F4 and M1 have already had a small argument the day before about M1 always agreeing to short-term night shifts and working overtime. Therefore, she is angry that he is cancelling the long-planned visit to the restaurant again.
- **High intensity:** Beforehand, there was an almost daily argument between F4 and M1 about M1 working too much and not resisting overtime and night shifts scheduled at short notice. The restaurant visit was planned by M1 as a reconciliation and was supposed to take place on their wedding day. Now F3 is bewildered and very angry that M1 is cancelling their evening together again.

**Practice Scene: Paradoxical Sarcasm (B15) – New House**

| Main Actor: **F1**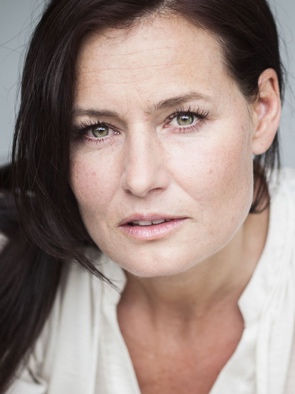 | Communication Partner: M2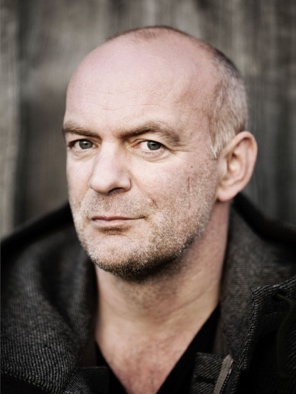 |
| --- | --- |

**Background Information**

- F1 and M2 have been married for 20 years and live together in an apartment.
- Simone is M2's cousin and lives in a neighbouring village. She is currently building a single-family house with her husband.

**Intensities**

- **Low intensity:** F1 generally likes Simone. She has no problem with her and is not jealous that Simone is able to build a new house. However, F1 thinks that Simone has questionable taste and no sense of aesthetics. Therefore, she makes fun of Simone's choice of colour.
- **Medium intensity:** F1 does not like Simone very much, so she ridicules Simone's new house.
- **High intensity:** F1 cannot stand Simone. She is jealous that Simone can afford to build her own home. F1 thinks that Simone does not deserve to own a home. In order to depreciate Simone, she viciously gossips about her at every opportunity.
- M2 also thinks that Simone did not choose a good colour for her house. However, M2 finds his wife is gossiping excessively. He is not interested in his cousin and her house. For this reason, he is not interested in the "gossip" with his wife and wants to end it as quickly as possible with the sentence "maybe she is colour-blind" so that he can continue reading the newspaper in peace. M2's reactions hardly change across all intensities.

# Appendix F

## Table F1: Scene H#1

### Test Questions of the BASIT-ToM and TASIT-SIM with their Corresponding Correct Answers, Types and Orders of Theory of Mind

| BASIT-ToM | | | | |  | TASIT-SIM | | | | |
| --- | --- | --- | --- | --- | --- | --- | --- | --- | --- | --- |
| Question  (German) | Question  (English) | ToM type | ToM order | Correct  answer |  | Question | ToM type | ToM order | Correct  answer | Question ID |
| Möchte er ihr vermitteln, dass sie unterschiedlicher Meinung sind? | Does he want to convey to her that they disagree? | cogn | 2^nd^ | No |  | Is Michael agreeing with Ruth about the movie? | cogn | 2^nd^ | yes | a |
| War er von dem Theaterstück gelangweilt? | Was he bored by the play? | aff | 1^st^ | No |  | Is he openly pleased that he saw the movie? | aff | 1^st^ | yes | b |
| Versucht er ihr zu sagen, dass er die Schauspieler schlecht findet? | Is he trying to say he thought the actors were bad? | aff | 1^st^ | No |  | Is he trying to say he thought the actors were good? | aff | 1^st^ | yes | c |

| BASIT-ToM | | | | |  | TASIT-SIM | | | | |
| --- | --- | --- | --- | --- | --- | --- | --- | --- | --- | --- |
| Question  (German) | Question  (English) | ToM type | ToM order | Correct  answer |  | Question | ToM type | ToM order | Correct  answer | Question ID |
| Denkt er, dass ihr das Theaterstück gefallen hat? | Does he think she enjoyed the play? | aff | 2^nd^ | yes |  | Does he think the movie was bad? | aff | 1^st^ | no | d |

| Ist er in sie verliebt? | Is he in love with her? | hyper |  | no |  |  |  |  |  | e |
| --- | --- | --- | --- | --- | --- | --- | --- | --- | --- | --- |

*Note.* BASIT-ToM = Basel Version of the Awareness of Social Inference Test – Theory of Mind; TASIT-SIM = The Awareness of Social Inference Test – Social Inference Minimal;

cogn = cognitive Theory of Mind; aff = affective Theory of Mind, hyper = hypermentalization.

## Table F2: Scene H#2

### Test Questions of the BASIT-ToM and TASIT-SIM with their Corresponding Correct Answers, Types and Orders of Theory of Mind

| BASIT-ToM | | | | |  | TASIT-SIM | | | | |
| --- | --- | --- | --- | --- | --- | --- | --- | --- | --- | --- |
| Question  (German) | Question  (English) | ToM type | ToM order | Correct  answer |  | Question | ToM type | ToM order | Correct  answer | Question ID |
| Möchte er ihr vermitteln, dass er ihr für die Organisation der Eintrittskarten dankbar ist? | Is Michael trying to show he appreciates Gary getting the tickets? | aff | 1^st^ | yes |  | Is Michael trying to show he appreciates Gary getting the tickets? | aff | 1^st^ | yes | a |
| Freut er sich, dass sie ihm die Eintrittskarten organisiert hat? | Is he happy that she organised the tickets for him | aff | 1^st^ | yes |  | Is Michael annoyed Gary got him the tickets? | aff | 1^st^ | no | b |
|  |  |  |  |  |  |  |  |  |  |  |
| Versucht er ihr zu sagen, dass er sich über die Eintrittskarten freut? | Is he trying to say he’s pleased about the tickets? | aff | 1^st^ | yes |  | Is he trying to say he’s pleased about the tickets? | aff | 1^st^ | yes | c |

| BASIT-ToM | | | | |  | TASIT-SIM | | | | |
| --- | --- | --- | --- | --- | --- | --- | --- | --- | --- | --- |
| Question  (German) | Question  (English) | ToM type | ToM order | Correct  answer |  | Question | ToM type | ToM order | Correct  answer | Question ID |
| Denkt sie, dass er kein Interesse an den Eintrittskarten hat? | Does she think that he is not interested in the tickets? | aff | 2^nd^ | no |  | By the end of the scene, does Gary think Michael wants to go? | cogn | 2^nd^ | yes | d |
|  |  |  |  |  |  |  |  |  |  |  |
| Möchte sie sich seine Freundschaft erkaufen? | Does he want to bribe him to become friends with him? | hyper |  | no |  |  |  |  |  | e |

*Note.* BASIT-ToM = Basel Version of the Awareness of Social Inference Test – Theory of Mind; TASIT-SIM = The Awareness of Social Inference Test – Social Inference Minimal;

cogn = cognitive Theory of Mind; aff = affective Theory of Mind, hyper = hypermentalization.

## Table F3: Scene H#3

### Test Questions of the BASIT-ToM and TASIT-SIM with their Corresponding Correct Answers, Types and Orders of Theory of Mind

| BASIT-ToM | | | | |  | TASIT-SIM | | | | |
| --- | --- | --- | --- | --- | --- | --- | --- | --- | --- | --- |
| Question  (German) | Question  (English) | ToM type | ToM order | Correct  answer |  | Question | ToM type | ToM order | Correct  answer | Question ID |
| Möchte sie, dass er sich geschätzt fühlt? | Is Ruth trying to make Michael feel appreciated? | aff | 2^nd^ | yes |  | Is Ruth trying to make Michael feel appreciated? | aff | 2^nd^ | yes | a |
|  |  |  |  |  |  |  |  |  |  |  |
| Ärgert sie sich über ihn? | Is she annoyed with him? | aff | 1^st^ | no |  | Is she annoyed with him? | aff | 1^st^ | no | b |
| Versucht sie ihm zu sagen, dass er ihr keine Hilfe war? | Is she trying to tell him that he has not been a big help? | cogn | 1^st^ | no |  | Is he trying to say that he has been a big help? | cogn | 1^st^ | yes | c |
|  |  |  |  |  |  |  |  |  |  |  |
| Denkt er, dass sie möchte, dass er am nächsten Tag wiederkommt? | Does he think she wants him to come back the next day? | cogn | 2^nd^ | yes |  | Is she annoyed with him? | aff | 1^st^ | no | d |

| BASIT-ToM | | | | |  | TASIT-SIM | | | | |
| --- | --- | --- | --- | --- | --- | --- | --- | --- | --- | --- |
| Question  (German) | Question  (English) | ToM type | ToM order | Correct  answer |  | Question | ToM type | ToM order | Correct  answer | Question ID |
| Bedankt sie sich bei ihm, damit er ihr am nächsten Tag erneut hilft? | Does she thank him so that he will help her again the next day? | hyper |  | no |  |  |  |  |  | e |

*Note.* BASIT-ToM = Basel Version of the Awareness of Social Inference Test – Theory of Mind; TASIT-SIM = The Awareness of Social Inference Test – Social Inference Minimal;

cogn = cognitive Theory of Mind; aff = affective Theory of Mind, hyper = hypermentalization.

## Table F4: Scene H#4

### Test Questions of the BASIT-ToM and TASIT-SIM with their Corresponding Correct Answers, Types and Orders of Theory of Mind

| BASIT-ToM | | | | |  | TASIT-SIM | | | | |
| --- | --- | --- | --- | --- | --- | --- | --- | --- | --- | --- |
| Question  (German) | Question  (English) | ToM type | ToM order | Correct  answer |  | Question | ToM type | ToM order | Correct  answer | Question ID |
| Möchte sie ihm vermitteln, dass er kaum Chancen auf eine Beförderung hat? | Is she trying to convey to him that he has little chance of being promoted? | cogn | 1^st^ | no |  | Is Ruth sending Michael up about his chances of a promotion? | cogn | 1^st^ | yes | a |
|  |  |  |  |  |  |  |  |  |  |  |
| Würde sie ihm die Beförderung missgönnen? | Would she begrudge him the promotion? | aff | 1^st^ | no |  | Would she like him to get the promotion? | aff | 1^st^ | yes | b |
| Versucht sie ihm zu sagen, dass er gut gearbeitet hat? | Is she trying to say he has worked really well? | cogn | 1^st^ | yes |  | Is she trying to say he has worked really well? | cogn | 1^st^ | yes | c |

| BASIT-ToM | | | | |  | TASIT-SIM | | | | |
| --- | --- | --- | --- | --- | --- | --- | --- | --- | --- | --- |
| Question  (German) | Question  (English) | ToM type | ToM order | Correct  answer |  | Question | ToM type | ToM order | Correct  answer | Question ID |
| Denkt er, dass sie ihre Komplimente ernst meint? | Does he think she is serious about her compliments? | cogn | 2^nd^ | yes |  | Does she think he deserves a promotion? | aff | 1^st^ | yes | d |
|  |  |  |  |  |  |  |  |  |  |  |
| Lobt sie ihn, um sich berufliche Vorteile zu verschaffen? | Is she praising him to gain a professional advantage? | hyper |  | no |  |  |  |  |  | e |

*Note.* BASIT-ToM = Basel Version of the Awareness of Social Inference Test – Theory of Mind; TASIT-SIM = The Awareness of Social Inference Test – Social Inference Minimal;

cogn = cognitive Theory of Mind; aff = affective Theory of Mind, hyper = hypermentalization.

## Table F5: Scene sS#1

### Test Questions of the BASIT-ToM and TASIT-SIM with their Corresponding Correct Answers, Types and Orders of Theory of Mind

| BASIT-ToM | | | | |  | TASIT-SIM | | | | |
| --- | --- | --- | --- | --- | --- | --- | --- | --- | --- | --- |
| Question  (German) | Question  (English) | ToM type | ToM order | Correct  answer |  | Question | ToM type | ToM order | Correct  answer | Question ID |
| Möchte sie, dass er sich trotz seiner Absage gut fühlt? | Is he trying to make him feel OK despite his cancellation? | aff | 2^nd^ | no |  | Is Ruth trying to make Michael feel OK? | aff | 2^nd^ | no | a |
|  |  |  |  |  |  |  |  |  |  |  |
| Ärgert sie sich über seine Absage? | Is she annoyed by his cancellation? | aff | 1^st^ | yes |  | Is she annoyed with him? | aff | 1^st^ | yes | b |
| Versucht sie ihm zu sagen, dass seine Absage ihr Probleme verursacht? | Is she trying to tell him that his cancellation is causing her problems? | cogn | 1^st^ | yes |  | Is she trying to say that he is causing a big problem? | cogn | 1^st^ | yes | c |

| BASIT-ToM | | | | |  | TASIT-SIM | | | | |
| --- | --- | --- | --- | --- | --- | --- | --- | --- | --- | --- |
| Question  (German) | Question  (English) | ToM type | ToM order | Correct  answer |  | Question | ToM type | ToM order | Correct  answer | Question ID |
| Denkt er, dass seine Absage für sie kein Problem darstellt? | Does he think that his cancellation is not a problem for her? | cogn | 2^nd^ | no |  | Does she believe he is too busy to take the class? | cogn | 2^nd^ | no | d |
|  |  |  |  |  |  |  |  |  |  |  |
| Glaubt sie, dass er ihr absagt, weil er ihr schaden möchte? | Does she think he is cancelling on her because he wants to harm her? | hyper |  | no |  |  |  |  |  | e |

*Note.* BASIT-ToM = Basel Version of the Awareness of Social Inference Test – Theory of Mind; TASIT-SIM = The Awareness of Social Inference Test – Social Inference Minimal;

cogn = cognitive Theory of Mind; aff = affective Theory of Mind, hyper = hypermentalization.

## Table F6: Scene sS#2

### Test Questions of the BASIT-ToM and TASIT-SIM with their Corresponding Correct Answers, Types and Orders of Theory of Mind

| BASIT-ToM | | | | |  | TASIT-SIM | | | | |
| --- | --- | --- | --- | --- | --- | --- | --- | --- | --- | --- |
| Question  (German) | Question  (English) | ToM type | ToM order | Correct  answer |  | Question | ToM type | ToM order | Correct  answer | Question ID |
| Möchte er weitere Verwandte einladen? | Does he want to invite other relatives? | cogn | 1^st^ | no |  | Is he seriously suggesting they inivite other family members? | cogn | 1^st^ | no | a |
|  |  |  |  |  |  |  |  |  |  |  |
| Ärgert er sich über sie? | Is he annoyed with her? | aff | 1^st^ | yes |  | Is he annoyed with her? | aff | 1^st^ | yes | b |
|  |  |  |  |  |  |  |  |  |  |  |
| Versucht er ihr zu sagen, dass ihre Verwandten mitkommen sollen? | Is he trying to say he want her relatives to come? | cogn | 1^st^ | no |  | Is he trying to say he doesn’t want her relatives to come? | cogn | 1^st^ | yes | c |
|  |  |  |  |  |  |  |  |  |  |  |
| Denkt sie, er möchte, dass ihre Verwandten mitkommen? | Does she think he wants her relatives to come? | cogn | 2^nd^ | no |  | Does she think he wants her relatives to come? | cogn | 2^nd^ | no | d |

| BASIT-ToM | | | | |  | TASIT-SIM | | | | |
| --- | --- | --- | --- | --- | --- | --- | --- | --- | --- | --- |
| Question  (German) | Question  (English) | ToM type | ToM order | Correct  answer |  | Question | ToM type | ToM order | Correct  answer | Question ID |
| Denkt er, dass sie den Besuch ihrer Schwester anspricht, um ihn zu provozieren? | Does he think she mentioning her sister's visit to provoke him? | hyper |  | no |  |  |  |  |  | e |

*Note.* BASIT-ToM = Basel Version of the Awareness of Social Inference Test – Theory of Mind; TASIT-SIM = The Awareness of Social Inference Test – Social Inference Minimal;

cogn = cognitive Theory of Mind; aff = affective Theory of Mind, hyper = hypermentalization.

## Table F7: Scene sS#3

### Test Questions of the BASIT-ToM and TASIT-SIM with their Corresponding Correct Answers, Types and Orders of Theory of Mind

| BASIT-ToM | | | | |  | TASIT-SIM | | | | |
| --- | --- | --- | --- | --- | --- | --- | --- | --- | --- | --- |
| Question  (German) | Question  (English) | ToM type | ToM order | Correct  answer |  | Question | ToM type | ToM order | Correct  answer | Question ID |
| Möchte er, dass sie sich schlecht fühlt? | Is he trying to make her feel bad? | aff | 2^nd^ | no |  | Is Gary trying to make Ruth feel bad? | aff | 2^nd^ | no | a |
|  |  |  |  |  |  |  |  |  |  |  |
| Ist er wütend auf sie? | Is he angry with her? | aff | 1^st^ | no |  | Is he angry with her? | aff | 1^st^ | no | b |
|  |  |  |  |  |  |  |  |  |  |  |
| Versucht er ihr zu sagen, dass sie gut aussieht? | Is he trying to say that she looks good? | cogn | 1^st^ | yes |  | Is he trying to say that she looks ok? | cogn | 1^st^ | yes | c |
|  |  |  |  |  |  |  |  |  |  |  |
| Denkt sie, dass er scherzt? | Does she think he is joking with her? | cogn | 2^nd^ | yes |  | Does she think he is joking with her? | cogn | 2^nd^ | yes | d |

| BASIT-ToM | | | | |  | TASIT-SIM | | | | |
| --- | --- | --- | --- | --- | --- | --- | --- | --- | --- | --- |
| Question  (German) | Question  (English) | ToM type | ToM order | Correct  answer |  | Question | ToM type | ToM order | Correct  answer | Question ID |
| Ist er in sie verliebt? | Is he in love with her? | hyper |  | no |  |  |  |  |  | e |

*Note.* BASIT-ToM = Basel Version of the Awareness of Social Inference Test – Theory of Mind; TASIT-SIM = The Awareness of Social Inference Test – Social Inference Minimal;

cogn = cognitive Theory of Mind; aff = affective Theory of Mind, hyper = hypermentalization.

## Table F8: Scene sS#4

### Test Questions of the BASIT-ToM and TASIT-SIM with their Corresponding Correct Answers, Types and Orders of Theory of Mind

| BASIT-ToM | | | | |  | TASIT-SIM | | | | |
| --- | --- | --- | --- | --- | --- | --- | --- | --- | --- | --- |
| Question  (German) | Question  (English) | ToM type | ToM order | Correct  answer |  | Question | ToM type | ToM order | Correct  answer | Question ID |
| Möchte sie ihm vermitteln, dass sein Hemd schrecklich aussieht? | Is she trying to make him understand that his shirt looks awful? | cogn | 1^st^ | yes |  | Is Ruth reassuring Gary that the shirt is nice? | cogn | 1^st^ | no | a |
|  |  |  |  |  |  |  |  |  |  |  |
| Ärgert sie sich darüber, dass er das Hemd trägt? | Is she annoyed that he is wearing the shirt? | aff | 1^st^ | yes |  | Is she happy for him to wear the shirt? | aff | 1^st^ | no | b |
|  |  |  |  |  |  |  |  |  |  |  |
| Versucht sie ihm zu sagen, dass sie das Hemd schön findet? | Is she trying to say the shirt is beautiful? | cogn | 1^st^ | no |  | Is she trying to say the shirt is awful? | cogn | 1^st^ | yes | c |

| BASIT-ToM | | | | |  | TASIT-SIM | | | | |
| --- | --- | --- | --- | --- | --- | --- | --- | --- | --- | --- |
| Question  (German) | Question  (English) | ToM type | ToM order | Correct  answer |  | Question | ToM type | ToM order | Correct  answer | Question ID |
| Denkt er, dass sie das Hemd schrecklich findet? | Does he think she finds the shirt awful? | cogn | 2^nd^ | yes |  | Does she think the shirt is OK? | cogn | 1^st^ | no | d |
|  |  |  |  |  |  |  |  |  |  |  |
| Denkt sie, dass er das Hemd anziehen möchte, um sie zu provozieren? | Is she thinking he wants to wear the shirt to provoke her? | hyper |  | no |  |  |  |  |  | e |

*Note.* BASIT-ToM = Basel Version of the Awareness of Social Inference Test – Theory of Mind; TASIT-SIM = The Awareness of Social Inference Test – Social Inference Minimal;

cogn = cognitive Theory of Mind; aff = affective Theory of Mind, hyper = hypermentalization.

## Table F9: Scene pS#1

### Test Questions of the BASIT-ToM and TASIT-SIM with their Corresponding Correct Answers, Types and Orders of Theory of Mind

| BASIT-ToM | | | | |  | TASIT-SIM | | | | |
| --- | --- | --- | --- | --- | --- | --- | --- | --- | --- | --- |
| Question  (German) | Question  (English) | ToM type | ToM order | Correct  answer |  | Question | ToM type | ToM order | Correct  answer | Question ID |
| Möchte sie ihm vermitteln, dass das Schreiben des Berichts aufwendig war? | Is she trying to convey to him that writing the report took a lot of work? | cogn | 1^st^ | yes |  | Is Ruth denying the report took a lot of work? | cogn | 1^st^ | no | a |
|  |  |  |  |  |  |  |  |  |  |  |
| Ist sie unzufrieden, weil sie am Wochenende arbeiten musste? | Does Ruth seem unhappy about working all weekend? | aff | 1^st^ | yes |  | Does Ruth seem happy about working all weekend? | aff | 1^st^ | no | b |
|  |  |  |  |  |  |  |  |  |  |  |
| Versucht sie ihm zu sagen, dass sie ein  anstrengendes Wochenende hatte? | Is she trying to tell him that she had an  exhausting weekend? | cogn | 1^st^ | yes |  | Is she trying to say she had a lazy, relaxing weekend? | cogn | 1^st^ | no | c |

| BASIT-ToM | | | | |  | TASIT-SIM | | | | |
| --- | --- | --- | --- | --- | --- | --- | --- | --- | --- | --- |
| Question  (German) | Question  (English) | ToM type | ToM order | Correct  answer |  | Question | ToM type | ToM order | Correct  answer | Question ID |
| Denkt er, dass es für sie in Ordnung war, am Wochenende zu arbeiten? | Does he think it was OK for her working all weekend? | aff | 2^nd^ | yes / no* |  | Does Michael think she took it easy on the weekend? | cogn | 1^st^ | no | d |
|  |  |  |  |  |  |  |  |  |  |  |
| Spricht er sie auf den Bericht an, um sie zu provozieren? | Is he talking to her about the report to provoke her? | hyper |  | no |  |  |  |  |  | e |

*Note.* BASIT-ToM = Basel Version of the Awareness of Social Inference Test – Theory of Mind; TASIT-SIM = The Awareness of Social Inference Test – Social Inference Minimal;

cogn = cognitive Theory of Mind; aff = affective Theory of Mind, hyper = hypermentalization.

*correct response = yes (low intensity), correct response = no (medium and high intensities)

## Table F10: Scene pS#2

### Test Questions of the BASIT-ToM and TASIT-SIM with their Corresponding Correct Answers, Types and Orders of Theory of Mind

| BASIT-ToM | | | | |  | TASIT-SIM | | | | |
| --- | --- | --- | --- | --- | --- | --- | --- | --- | --- | --- |
| Question  (German) | Question  (English) | ToM type | ToM order | Correct  answer |  | Question | ToM type | ToM order | Correct  answer | Question ID |
| Möchte er sich negativ über die Ohrringe äussern? | Is he commenting negatively on the earrings? | cogn | 1^st^ | yes |  | Is Michael being complimentary about the dress? | cogn | 1^st^ | no | a |
|  |  |  |  |  |  |  |  |  |  |  |
| Amüsiert er sich über die Qualität der Ohrringe? | Is she making fun of the quality of the earrings? | aff | 1^st^ | yes |  | Does he like the dress? | aff | 1^st^ | no | b |
|  |  |  |  |  |  |  |  |  |  |  |
| Versucht er ihr zu sagen, dass die Ohrringe hochwertig aussehen? | Is he trying to tell her that the earrings look high quality? | cogn | 1^st^ | no |  | Is he trying to say the dress looks cheap? | cogn | 1^st^ | yes | c |
|  |  |  |  |  |  |  |  |  |  |  |
| Denkt er, dass ihr die Ohrringe gefallen? | Does he think she likes the earrings? | aff | 2^nd^ | no |  | Does he think Ruth’s sister paid a lot for the dress? | cogn | 1^st^ | no | d |

| BASIT-ToM | | | | |  | TASIT-SIM | | | | |
| --- | --- | --- | --- | --- | --- | --- | --- | --- | --- | --- |
| Question  (German) | Question  (English) | ToM type | ToM order | Correct  answer |  | Question | ToM type | ToM order | Correct  answer | Question ID |
| Können sie die Schwester nicht ausstehen? | Can they not stand the sister? | hyper |  | no |  |  |  |  |  | e |

*Note.* BASIT-ToM = Basel Version of the Awareness of Social Inference Test – Theory of Mind; TASIT-SIM = The Awareness of Social Inference Test – Social Inference Minimal;

cogn = cognitive Theory of Mind; aff = affective Theory of Mind, hyper = hypermentalization.

## Table F11: Scene pS#3

### Test Questions of the BASIT-ToM and TASIT-SIM with their Corresponding Correct Answers, Types and Orders of Theory of Mind

| BASIT-ToM | | | | |  | TASIT-SIM | | | | |
| --- | --- | --- | --- | --- | --- | --- | --- | --- | --- | --- |
| Question  (German) | Question  (English) | ToM type | ToM order | Correct  answer |  | Question | ToM type | ToM order | Correct  answer | Question ID |
| Möchte er sich negativ über das Essen äussern? | Is he commenting negatively on the meal? | cogn | 1^st^ | no |  | Is Gary trying to compliment Ruth on the meal? | cogn | 1^st^ | yes | a |
|  |  |  |  |  |  |  |  |  |  |  |
| Hat er das Essen genossen? | Did he enjoy the meal? | aff | 1^st^ | yes |  | Did he enjoy the meal? | aff | 1^st^ | yes | b |
|  |  |  |  |  |  |  |  |  |  |  |
| Versucht er ihr zu sagen, dass das Essen schlecht geschmeckt hat? | Is he trying to tell her that the meal tasted awful? | cogn | 1^st^ | no |  | Is he trying to say that the meal tasted awful? | aff | 1^st^ | no | c |
|  |  |  |  |  |  |  |  |  |  |  |
| Denkt sie, dass ihm das Essen schlecht geschmeckt hat? | Does she think he did not like the meal? | cogn | 2^st^ | no |  | Does he think she is a good cook? | cogn | 1^st^ | yes | d |

| BASIT-ToM | | | | |  | TASIT-SIM | | | | |
| --- | --- | --- | --- | --- | --- | --- | --- | --- | --- | --- |
| Question  (German) | Question  (English) | ToM type | ToM order | Correct  answer |  | Question | ToM type | ToM order | Correct  answer | Question ID |
| Ist er in sie verliebt? | Is he in love with her? | hyper |  | no |  |  |  |  |  | e |

*Note.* BASIT-ToM = Basel Version of the Awareness of Social Inference Test – Theory of Mind; TASIT-SIM = The Awareness of Social Inference Test – Social Inference Minimal;

cogn = cognitive Theory of Mind; aff = affective Theory of Mind, hyper = hypermentalization.

## Table F12: Scene pS#4

### Test Questions of the BASIT-ToM and TASIT-SIM with their Corresponding Correct Answers, Types and Orders of Theory of Mind

| BASIT-ToM | | | | |  | TASIT-SIM | | | | |
| --- | --- | --- | --- | --- | --- | --- | --- | --- | --- | --- |
| Question  (German) | Question  (English) | ToM type | ToM order | Correct  answer |  | Question | ToM type | ToM order | Correct  answer | Question ID |
| Möchte sie, dass er sich aufgrund seiner Absage schlecht fühlt? | Is she trying to make him feel bad about his cancellation? | aff | 2^nd^ | yes |  | Is Ruth trying to make Gary feel OK about cancelling? | aff | 2^nd^ | no | a |
|  |  |  |  |  |  |  |  |  |  |  |
| Ist sie von seiner Absage enttäuscht? | Is she disappointed by his cancellation? | aff | 1^st^ | yes |  | Is she happy to cancel? | aff | 1^st^ | no | b |
|  |  |  |  |  |  |  |  |  |  |  |
| Versucht sie ihm zu sagen, dass sie froh ist, an diesem Abend zu Hause bleiben zu können? | Is she trying to tell him that she is happy to stay at home that night? | aff | 1^st^ | no |  | Is she trying to say she wanted to go out that night? | cogn | 1^st^ | yes | c |

| BASIT-ToM | | | | |  | TASIT-SIM | | | | |
| --- | --- | --- | --- | --- | --- | --- | --- | --- | --- | --- |
| Question  (German) | Question  (English) | ToM type | ToM order | Correct  answer |  | Question | ToM type | ToM order | Correct  answer | Question ID |
| Denkt er, dass sie froh ist, an diesem Abend zu Hause bleiben zu können? | Does he think she is happy to stay at home that night? | aff | 2^nd^ | no |  | *By the end of the scene:* Does he think she’s upset about cancelling dinner? | aff | 2^nd^ | yes | d |
|  |  |  |  |  |  |  |  |  |  |  |
| Glaubt sie, dass er eine Affäre hat und nur vorgibt nachts zu arbeiten? | Does she think he is cheating on her and just pretending to work nights? | hyper |  | no |  |  |  |  |  | e |

*Note.* BASIT-ToM = Basel Version of the Awareness of Social Inference Test – Theory of Mind; TASIT-SIM = The Awareness of Social Inference Test – Social Inference Minimal;

cogn = cognitive Theory of Mind; aff = affective Theory of Mind, hyper = hypermentalization.

## Table F13: Scene Practice(pS)

### Test Questions of the BASIT-ToM and TASIT-SIM with their Corresponding Correct Answers, Types and Orders of Theory of Mind

| BASIT-ToM | | | | |  | TASIT-SIM | | | | |
| --- | --- | --- | --- | --- | --- | --- | --- | --- | --- | --- |
| Question  (German) | Question  (English) | ToM type | ToM order | Correct  answer |  | Question | ToM type | ToM order | Correct  answer | Question ID |
| Möchte sie sich positiv über Simones Haus äussern? | Is she being complementary about Simone’s house? | cogn | 1^st^ | no |  | Is Ruth being complementary about her friend’s house? | cogn | 1^st^ | no | a |
|  |  |  |  |  |  |  |  |  |  |  |
| Gefällt ihr die Farbgebung des Hauses? | Does she like the color scheme of the house? | aff | 1^st^ | no |  | Does she disapprove of the colour scheme? | aff | 1^st^ | yes | b |
|  |  |  |  |  |  |  |  |  |  |  |
| Versucht sie zu sagen, dass sie die Farbgebung des Hauses schrecklich findet? | Is she trying to say the color scheme of the house is dreadful? | cogn | 1^st^ | yes |  | Is she trying to say the colour scheme is dreadful? | cogn | 1^st^ | yes | c |
|  |  |  |  |  |  |  |  |  |  |  |
| Denkt er, dass ihr Simones neues Haus gefällt? | Does he think she likes Simone’s new house? | aff | 2^nd^ | no |  | Does she think her friend has good taste? | cogn | 1^st^ | nein | d |
| BASIT-ToM | | | | |  | TASIT-SIM | | | | |
| Question  (German) | Question  (English) | ToM type | ToM order | Correct  answer |  | Question | ToM type | ToM order | Correct  answer | Question ID |
| Können beide Simone nicht ausstehen? | Can they both not stand Simone? | hyper |  | nein |  |  |  |  |  | e |

*Note.* BASIT-ToM = Basel Version of the Awareness of Social Inference Test – Theory of Mind; TASIT-SIM = The Awareness of Social Inference Test – Social Inference Minimal;

cogn = cognitive Theory of Mind; aff = affective Theory of Mind, hyper = hypermentalization.

# Appendix G

## Table G1

### Main Differences between the BASIT-ToM and TASIT-SIM

|  | BASIT-ToM | TASIT-SIM |
| --- | --- | --- |
| Test length  Test versions    Number of scenes | One test form  (scenes from the TASIT-SIM forms A and B are included)  9 (3x3) scenes and one practice scene | Two parallel test forms, named A and B (form A has been used in the majority of published studies)  Form A: 15 (5x3) scenes  Form B: 15 (5x3) scenes  In total 30 scenes |
| Scenes  Distribution of actors    Gender distribution    Age of the actors    Interaction scenes  Script | Each actor portrays a message type only once.  Evenly across message types.  Either a male actor or a female actor portrays the message types.  Middle-aged actors  Communication partners react in a realistic manner  The names of the actors are omitted and replaced as "she" / "he” | Unevenly distributed across message types  Almost evenly across message types of the test scenes but across all scenes more male actors (61%)  Quite wide range of age, but rather actors in their twenties/thirties  Communication partners act in a neutral emotional state  Each actor bears a name which he/she keeps throughout all scenes |

|  | BASIT-ToM | TASIT-SIM |
| --- | --- | --- |
| Production design | Consistent, realistic settings: at home or at the office;  camera perspective and setting dimensions convey realism | Inconsistent and partly unrealistic settings (partly black background, office or living room setting); camera perspective and setting dimensions do not convey realism (e.g., mostly long shots) |
| Test questions | 5 test questions per each scene assigned to ToM types and orders  (i.e., 1^st^ order affective ToM: *n*=17, 1^st^ order cognitive ToM: *n*=17, 2^nd^ order affective ToM: *n*=10, 2^nd^ order cognitive ToM: *n*=8, hypermentalization: *n*=13)  Questions refer to scene’s main actor and his/her communication partner | 4 test questions per each scene divided based on content  (i.e., 1^st^ order affective ToM: *n*=18, 1^st^ order cognitive ToM: *n*=24, 2^nd^ order affective ToM: *n*=5, 2^nd^ order cognitive ToM: *n*=5)  Questions refer to the scene’s main actor |

*Note.* BASIT-ToM = Basel Version of the Awareness of Social Inference Test–Theory of Mind; TASIT-SIM = The Awareness of Social Inference Test–Social Inference Minimal; ToM = Theory of Mind

# Appendix H

## Document H1

### Details of the Programming and Data Storage

BASIT-ToM intensity versions scenes were implemented as part of a suit of tests. The order was as follows:

1. BASIT-ER intensity version scenes (masked)
2. BASIT-ToM intensity version scenes
3. Gender discrimination test (by use of facial stimuli)
4. Facial Emotional Intensity Recognition Test – Congruent (FEIRT-C) (Chiu et al., 2016)
5. Facial Emotion Intensity Recognition Test – Congruent and Incongruent (FEIRT-CIC) (Chiu et al., 2018)

These five tests were included in an application written in Python 2.7 using version 1.84.2 of the PsychoPy package (Pierce, 2007, 2009). The application was installed on a workstation running Ubuntu 16.04 LTS.

The pseudo-random selection and order of scenes shown to the participants was created beforehand using R: First, a random sample containing message type (“Honesty”, “Simple Sarcasm”, “Paradoxical Sarcasm”) exactly four times was created, with the constraint that no successive message type was equal. For each participant ID the order of the four scenes per message type was randomised. Then, the intensities were randomly distributed: The 240 first scenes of each message type were randomly splitted in three parts of 80 scenes with low, medium, and high intensity, respectively. For each intensity of the first scene (e.g., Honesty#1_low) the second scene of the given message type was then randomly splitted into two parts of 40 scenes with the two other intensities (Honesty#2_medium and Honesty#2_high). The intensity of the third scene was then fixed to the intensity that was not yet assigned to the first two scenes. For the three fourth scenes (one per message type), a similar mechanism was used: The 240 scenes of the first message types were randomly splitted in three parts of 80 scenes with low, medium, and high intensities. For each intensity of the first message type, the second message type was randomly splitted into two parts of 40 scenes with the two other intensities. The intensity of the last message type was fixed by the intensity not yet assigned to the first two message types. Lastly, the intensity of the practice scene shown to each participant at the beginning of the test was randomly assigned, such that each intensity of the practice scene was shown to 80 participants.

This resulted in:

- Each participant viewed the practice scene and 12 scenes (4 per message type),
- Consecutive scenes with different message types,
- Each participant viewed at least once a low intensity, a medium intensity, and a high intensity version scene per message type
- Each participant viewed four low intensity, four medium intensity, and four high intensity version scenes in total.
- Each intensity version scene was shown to 80 participants.
- The first scene was the practice scene, shown in each intensity to 80 participants, respectively.

Both, the sequence of the five questions [i.e., four types of questions, originating from The Awareness of Social Inference Test – Social Inference Minimal, (i.e., “doing”, “saying”, “thinking”, “feeling”) and the hypermentalization question] phrased for each scene, and the “Yes”-“No”-answers phrased for each scene, were also pseudo-randomly fixed using R. For each message type (“Honesty”, “Simple Sarcasm”, “Paradoxical Sarcasm”), the correct answers for the “doing”-, “saying-”, “thinking”- and “feeling”-questions were randomly fixed to 50% “Yes”- and 50% “No”-answers over all four scenes and five types of questions per message type. Notably, in the “Paradoxical Sarcasm” scene “Bericht” (PS#1), the correct answer on the “thinking” question depends on the intensity of the scene (“Yes” for low intensity, otherwise “No”). The correct answer for all “hypermentalization” questions was set to “No”. The order of the five questions was randomly determined for each scene (independent of the intensity).

The results were stored in to 240 separate files containing the different sequences of scenes shown to the individual participants, together with the five questions and correct answers for each scene.

The sequence of the 13 scenes (1 practice scene, four scenes of three-interaction type) for a specific participant was loaded into the application at start. The respective sequence of the scenes was allocated to the participant’s ID.

The test started with a screen summarizing the instructions to the participant. By clicking on the button “next”, the practice scene started. Afterwards, the participant was asked whether anything was still unclear regarding the test procedure. If not, the participant started the test by clicking on the button “next”. In the following, the 12 scenes were shown to the participant in the prepared pseudo-randomized order. After each scene, the participants were asked to answer the five Yes/No questions. The participants were shown a message that the test was completed, before the application started the next test.

Test results were saved as .csv file and transferred to a secure cloud server provided by the Clinical Trial Unit of the University Hospital Basel (UHBS), backed up by the IT Department of the UHBS.

**References:**

- masked. The Basel Version of the Awareness of Social Inference Test – Emotion Recognition (BASIT-ER): Preliminary Validation Analyses in Healthy Adults.
- Chiu, I., Piguet, O., Diehl-Schmid, J., Riedl, L., Beck, J., Leyhe, T., . . . Sollberger, M. (2016). Dissociation in Rating Negative Facial Emotions between Behavioral Variant Frontotemporal Dementia and Major Depressive Disorder. Am J Geriatr Psychiatry, 24(11), 1017-1027. doi:10.1016/j.jagp.2016.06.011
- Chiu, I., Piguet, O., Diehl-Schmid, J., Riedl, L., Beck, J., Leyhe, T., . . . Sollberger, M. (2018). Facial Emotion Recognition Performance Differentiates Between Behavioral Variant Frontotemporal Dementia and Major Depressive Disorder. J Clin Psychiatry, 79(1). doi:10.4088/JCP.16m11342
- Peirce J. W. (2009). Generating stimuli for neuroscience using PsychoPy. *Frontiers in Neuroinformatics*, 2 (10), 1-8. doi:10.3389/neuro.11.010.2008
- Peirce, J. W. (2007). PsychoPy - Psychophysics software in Python. *Journal of Neuroscience Methods*, 162 (1-2):8-13 doi:10.1016/j.jneumeth.2006.11.017

# Appendix I

## Figure I1

Exemplary Representation of the BASIT-ToM Computer-Based Application Process


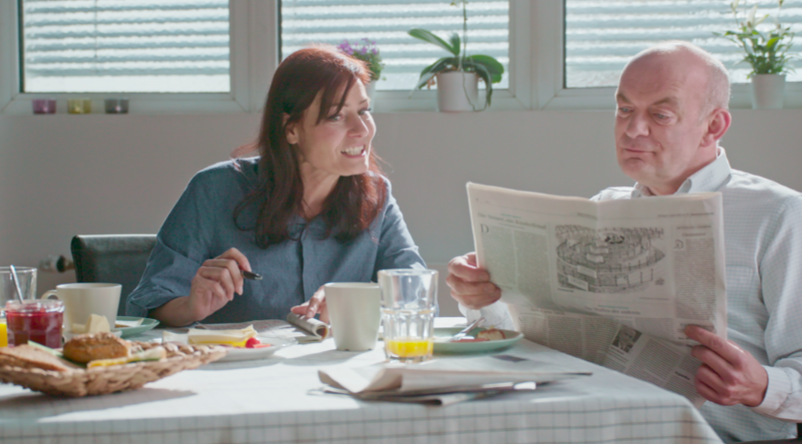


continue

replay

Is Ruth being complementary about Simone’s hous?

Is he trying to say the color scheme is dreadful?

Does she think Simone has good taste?

Does she disapprove of the color scheme?

Can they not stand Simone?

*Note.* The square represents the scene and the five circles the five questions. By clicking the black square, participants could rewatch the scene. By clicking a black circle, participants could reread the respective question and, if wanted, modify their answer.

# Appendix J

## Table J.1: Item Characteristic Curves of the Intensity Version Scenes for the Message Type Honesty

| Intensity versions fitting the Rasch Model | Number of ToM questions for which the correct response probability is in the required range (0.5-0.8) in subjects with medium ToM abilities | Item characteristic curves of the ToM and hyperToM questions of the intensity version fitting the Rasch model |
| --- | --- | --- |
| **H#1_low** | **2** |  |

| Intensity versions fitting the Rasch model | Number of ToM questions for which the correct response probability is in the required range (0.5-0.8) in subjects with medium ToM abilities | Item characteristic curves of the ToM and hyperToM questions of the intensity version fitting the Rasch model |
| --- | --- | --- |
| H#1_medium | 1 |  |

| Intensity versions fitting the Rasch model | Number of ToM questions for which the correct response probability is in the required range (0.5-0.8) in subjects with medium ToM abilities | Item characteristic curves of the ToM and hyperToM questions of the intensity version fitting the Rasch model |
| --- | --- | --- |
| H#1_high | 1 |  |

| Intensity versions fitting the Rasch model | Number of ToM questions for which the correct response probability is in the required range (0.5-0.8) in subjects with medium ToM abilities | Item characteristic curves of the ToM and hyperToM questions of the intensity version fitting the Rasch model |
| --- | --- | --- |
| **H#2_low** | **4** |  |

| Intensity versions fitting the Rasch model | Number of ToM questions for which the correct response probability is in the required range (0.5-0.8) in subjects with medium ToM abilities | Item characteristic curves of the ToM and hyperToM questions of the intensity version fitting the Rasch model |
| --- | --- | --- |
| H#3_low | 0 |  |

| Intensity versions fitting the Rasch model | Number of ToM questions for which the correct response probability is in the required range (0.5-0.8) in subjects with medium ToM abilities | Item characteristic curves of the ToM and hyperToM questions of the intensity version fitting the Rasch model |
| --- | --- | --- |
| **H#4_medium** | **1** |  |

*Note*. ToM = Theory of Mind. Scene code consists of a letter, representing the message type (i.e., H=Honesty), the scene number, and the portrayed intensity (i.e., low, medium, high). Item characteristic curves of the hyperToM question is only depicted in presence of a model fit. Question code consists of abbreviations of type (i.e., cogn = cognitive ToM, aff = affective ToM, hyperToM = hypermentalization) and order (i.e., 1 = first-order ToM, 2 = second-order ToM) of ToM, and question’s identification letter (a-d = ToM questions, e = hypermentalization question). Intensities versions selected for the BASIT-ToM are printed in bold.

## Table J.2: Item Characteristic Curves of the Intensity Version Scenes for Message Type Paradoxical Sarcasm

| Intensity versions fitting the Rasch model | Number of ToM questions for which the correct response probability is in the required range (0.5-0.8) in subjects with medium ToM abilities | Item characteristic curves of the ToM and hyperToM questions of the intensity version fitting the Rasch model |
| --- | --- | --- |
| **pS#1_low** | **4** |  |

| Intensity versions fitting the Rasch model | Number of ToM questions for which the correct response probability is in the required range (0.5-0.8) in subjects with medium ToM abilities | Item characteristic curves of the ToM and hyperToM questions of the intensity version fitting the Rasch model |
| --- | --- | --- |
| pS#1_medium | 1 |  |

| Intensity versions fitting the Rasch model | Number of ToM questions for which the correct response probability is in the required range (0.5-0.8) in subjects with medium ToM abilities | Item characteristic curves of the ToM and hyperToM questions of the intensity version fitting the Rasch model | |
| --- | --- | --- | --- |
| **pS#2_low** | **4** |  |  |

| Intensity versions fitting the Rasch model | Number of ToM questions for which the correct response probability is in the required range (0.5-0.8) in subjects with medium ToM abilities | Item characteristic curves of the ToM and hyperToM questions of the intensity version fitting the Rasch model |
| --- | --- | --- |
| **pS#4_low^†^** | **2** |  |

| Intensity versions fitting the Rasch model | | Number of ToM questions for which the correct response probability is in the required range (0.5-0.8) in subjects with medium ToM abilities | | Item characteristic curves of the ToM and hyperToM questions of the intensity version fitting the Rasch model | |
| --- | --- | --- | --- | --- | --- |
| **Practice(pS)_low^‡^** | **4** | |  | |  |

| Intensity versions fitting the Rasch model | Number of ToM questions for which the correct response probability is in the required range (0.5-0.8) in subjects with medium ToM abilities | | | Item characteristic curves of the ToM and hyperToM questions of the intensity version fitting the Rasch model |  |
| --- | --- | --- | --- | --- | --- |
| Practice(pS)_medium | | 2 |  | | |

| Intensity versions fitting the Rasch model | Number of ToM questions for which the correct response probability is in the required range (0.5-0.8) in subjects with medium ToM abilities | Item characteristic curves of the ToM and hyperToM questions of the intensity version fitting the Rasch model | |  |
| --- | --- | --- | --- | --- |
| Practice(pS)_high | 3 | |  | |

*Note*. ToM = Theory of Mind. Scene code consists of a letter, representing the message type (i.e., pS = Paradoxical Sarcasm), the scene number, and the portrayed intensity (i.e., low, medium, high). Item characteristic curves of the hyperToM question is only depicted in presence of a model fit. Question code consists of abbreviations of type (i.e., cogn = cognitive ToM, aff = affective ToM, hyperToM = hypermentalization) and order (i.e., 1 = first-order ToM, 2 = second-order ToM) of ToM, and question’s identification letter (a-d = ToM questions, e = hypermentalization question). Intensities versions selected for the BASIT-ToM are printed in bold.

^†^pS#4_low will be used as practice scene in the Basel Version of The Awareness of Social Inference Test - Theory of Mind (BASIT-ToM).

^‡^Practice(pS)_low will be used as a test scene for Paradoxical Sarcasm of the BASIT-ToM

## Table J.3: Item Characteristic Curves of the Intensity Version Scenes for Message Type Simple Sarcasm

| Intensity versions fitting the Rasch model | Number of ToM questions for which the correct response probability is in the required range (0.5-0.8) in subjects with medium ToM abilities | Item characteristic curves of the ToM and hyperToM questions of the intensity version fitting the Rasch model |
| --- | --- | --- |
| **sS#1_low** | **3** |  |

| Intensity versions fitting the Rasch model | Number of ToM questions for which the correct response probability is in the required range (0.5-0.8) in subjects with medium ToM abilities | Item characteristic curves of the ToM and hyperToM questions of the intensity version fitting the Rasch model |
| --- | --- | --- |
| sS#1_high | 1 |  |

| Intensity versions fitting the Rasch model | Number of ToM questions for which the correct response probability is in the required range (0.5-0.8) in subjects with medium ToM abilities | Item characteristic curves of the ToM and hyperToM questions of the intensity version fitting the Rasch model | |
| --- | --- | --- | --- |
| **sS#2_low** | **1** |  |  |

| Intensity versions fitting the Rasch model | Number of ToM questions for which the correct response probability is in the required range (0.5-0.8) in subjects with medium ToM abilities | Item characteristic curves of the ToM and hyperToM questions of the intensity version fitting the Rasch model |
| --- | --- | --- |
| sS#2_high | 0 |  |

| Intensity versions fitting the Rasch model | Number of ToM questions for which the correct response probability is in the required range (0.5-0.8) in subjects with medium ToM abilities | Item characteristic curves of the ToM and hyperToM questions of the intensity version fitting the Rasch model | |
| --- | --- | --- | --- |
| sS#3_low | 2 |  |  |

| Intensity versions fitting the Rasch model | Number of ToM questions for which the correct response probability is in the required range (0.5-0.8) in subjects with medium ToM abilities | Item characteristic curves of the ToM and hyperToM questions of the intensity version fitting the Rasch model |
| --- | --- | --- |
| **sS#3_medium** | **3** |  |

| Intensity versions fitting the Rasch model | Number of ToM questions for which the correct response probability is in the required range (0.5-0.8) in subjects with medium ToM abilities | Item characteristic curves of the ToM and hyperToM questions of the intensity version fitting the Rasch model |
| --- | --- | --- |
| sS#3_high | 2 |  |

| Intensity versions fitting the Rasch model | Number of ToM questions for which the correct response probability is in the required range (0.5-0.8) in subjects with medium ToM abilities | Item characteristic curves of the ToM and hyperToM questions of the intensity version fitting the Rasch model | |
| --- | --- | --- | --- |
| sS#4_medium | 1 |  |  |

| Intensity versions fitting the Rasch model | Number of ToM questions for which the correct response probability is in the required range (0.5-0.8) in subjects with medium ToM abilities | Item characteristic curves of the ToM and hyperToM questions of the intensity version fitting the Rasch model | |
| --- | --- | --- | --- |
| sS#4_high | 0 |  |  |

*Note*. ToM = Theory of Mind. Scene code consists of a letter, representing the message type (i.e., sS = Simple Sarcasm), the scene number, and the portrayed intensity (i.e., low, medium, high). Item characteristic curves of the hyperToM question is only depicted in presence of a model fit. Question code consists of abbreviations of type (i.e., cogn = cognitive ToM, aff = affective ToM, hyperToM = hypermentalization) and order (i.e., 1 = first-order ToM, 2 = second-order ToM) of ToM, and question’s identification letter (a-d = ToM questions, e = hypermentalization question). Intensities versions selected for the BASIT-ToM are printed in bold.

**Appendix K**

**Table K.1: Number of times each selected intensity version scene was watched by participants, depicted by the percentages of the respective 80 participants**

| Scene | Number of times the scene was watched in percentages^†^ | | | |
| --- | --- | --- | --- | --- |
|  | 1 | 2 | 3 | 4 |
| H#1_low | 91% | 9% |  |  |
| H#2_low | 89% | 11% |  |  |
| H#4_medium | 89% | 11% |  |  |
| sS#1_low | 91% | 9% |  |  |
| sS#2_low | 88% | 13% |  |  |
| sS#3_medium | 91% | 9% |  |  |
| pS#1_low | 88% | 11% |  | 1% |
| pS#2_low | 58% | 36% | 6% |  |
| pS#4_low^‡^ | 78% | 23% |  |  |
| Practice (pS_low)^§^ | 71% | 25% | 4% |  |

Note. Scene code consists of letters (i.e., H = Honesty, pS = Paradoxical Sarcasm, sS = Simple Sarcasm), the respective scene numbers and the intensity level.

^†^Rounded percentages may not necessarily add up to 100%.

^‡^pS#4_low (scene #4 containing Paradoxical Sarcasm at low intensity) will be used as practice scene.

^§^Practice(pS)_low will be used as a test scene for Paradoxical Sarcasm.
